# Effect of Wearing a Novel Electronic Wearable Device on Hand Hygiene Compliance Among Health Care Workers: A Stepped-Wedge Cluster Randomized Clinical Trial

**DOI:** 10.1001/jamanetworkopen.2020.35331

**Published:** 2021-02-08

**Authors:** Daniela Pires, Angele Gayet-Ageron, Chloe Guitart, Yves-Alain Robert, Carolina Fankhauser, Ermira Tartari, Alexandra Peters, Funda Tymurkaynak, Simon Fourquier, Herve Soule, Rene Beuchat, Fernando Bellissimo-Rodrigues, Yves Martin, Walter Zingg, Didier Pittet

**Affiliations:** 1 Infection Control Programme and World Health Organization Collaborating Centre on Patient Safety—Infection Control & Improving Practices, University of Geneva Hospitals and Faculty of Medicine, Geneva, Switzerland; 2 Faculty of Health Sciences, University of Malta, Msida, Malta; 3 iQati, Sion, Switzerland; 4 Haute école du paysage, d’ingénierie et d’architecture de Genève (HEPIA), Geneva, Switzerland; 5 Social Medicine Department, Ribeirão Preto Medical School, University of São Paulo, Ribeirão Preto, São Paulo, Brazil

## Abstract

**Importance:**

Hand hygiene (HH) is essential to prevent hospital-acquired infections.

**Objective:**

To determine whether providing real-time feedback on a simplified HH action improves compliance with the World Health Organization’s “5 Moments” and the quality of the HH action.

**Design, Setting, and Participants:**

This open-label, cluster randomized, stepped-wedge clinical trial was conducted between June 1, 2017, and January 6, 2018 (with a follow-up in March 2018), in a geriatric hospital of the University of Geneva Hospitals, Switzerland. All 12 wards and 97 of 306 eligible health care workers (HCWs) volunteered to wear a novel electronic wearable device that delivered real-time feedback on duration of hand rubbing and application of a hand-sized customized volume of alcohol-based handrub (ABHR).

**Interventions:**

This study had 3 sequential periods: baseline (no device), transition (device monitoring without feedback), and intervention (device monitoring and feedback). The start of the transition period was randomly allocated based on a computer-generated block randomization.

**Main Outcomes and Measures:**

The primary outcome was HH compliance, according to the direct observation method during intervention as compared with baseline. Secondary outcomes included the volume of ABHR and duration of hand rubbing measured by the device during intervention as compared with transition.

**Results:**

All wards and respective HCWs were evenly assigned to group 1 (26 participants), 2 (22 participants), 3 (25 participants), or 4 (24 participants). Twelve HCWs did not fully complete the intervention but were included in the analysis. During 759 observation sessions, 6878 HH opportunities were observed. HH compliance at intervention (62.9%; 95% CI, 61.1%-64.7%) was lower than at baseline (66.6%; 95% CI, 64.8%-68.4%). After adjusting for covariates, HH compliance was not different between periods (odds ratio, 1.03; 95% CI, 0.75-1.42; *P* = .85). Days since study onset (OR, 0.997; 95% CI, 0.994-0.998; *P* < .001), older age (OR, 0.97; 95% CI, 0.95-0.99; *P* = .015), and workload (OR, 0.29; 95% CI, 0.20-0.41; *P* < .001) were independently associated with reduced HH compliance. The median (interquartile range) volume of ABHR and duration of hand rubbing in transition and intervention increased from 1.12 (0.76-1.68) mL to 1.71 (1.01-2.76) mL and from 6.5 (4.5-10.5) seconds to 8 (4.5-15.5) seconds, respectively. There were no serious adverse events.

**Conclusions and Relevance:**

The use of this device did not change HH compliance, but increased the duration of hand rubbing and volume of ABHR used by HCWs.

**Trial Registration:**

isrctn.org Identifier: ISRCTN25430066

## Key Points

**Question:**

Does providing real-time feedback on a simplified hand hygiene (HH) action with a novel electronic wearable device improve the compliance with the “5 Moments” and the quality of the HH action according to World Health Organization (WHO) guidelines?

**Findings:**

In this stepped-wedge, cluster randomized clinical trial involving 97 health care workers at the University of Geneva Hospitals, the device did not improve compliance with the “5 Moments” of HH. The volume of alcohol-based handrub and the duration of hand rubbing per HH increased after the activation of the feedback.

**Meaning:**

The use of the device did not change HH compliance, but improved quality of the HH action.

## Teaser

This stepped-wedge, cluster randomized clinical trial assessed the effectiveness of wearable electronic devices in improving health care worker compliance with World Health Organization guidelines for hand hygiene.

## Introduction

Health care–associated infections (HAI) and the spread of antimicrobial resistance are major public health concerns^1,2^ that are largely avoidable in health care by effective implementation of infection prevention and control best practices.^3,4^ The World Health Organization (WHO) recommends that performing hand hygiene (HH) correctly (“How to Handrub”) at the correct time (“5 Moments”) is the most effective measure to prevent HAI.^5,6^ The appropriate HH action is crucial to assure proper antimicrobial efficacy.^7,8,9^ Unfortunately, HH compliance remains suboptimal,^10^ and new strategies are needed to improve its implementation.

A number of studies suggest that the HH action, as endorsed by the WHO, could be simplified without compromising efficacy. Proposed changes include shortening duration of hand rubbing to 15 seconds (instead of 30 seconds)^11,12,13,14^ and changing the number or order of steps (“3 steps” or “fingertips-first” instead of the “6 step” technique).^15,16,17^ Additionally, standardization of the hand-size (“palm full”) concept recommended by the WHO has been proposed.^18,19^ It is hypothesized that these changes could lead to an increase in the quality of the HH action performed by health care workers (HCWs).^13,16^ In addition, some small-sized studies have suggested that a simplified HH action could also improve compliance with the “5 Moments.”^13,16^

Monitoring and feedback are essential parts of the WHO multimodal strategy to improve HH.^6^ However, direct observation and timely feedback are time-consuming, costly, and prone to bias.^20,21^ There has been a growing interest in the use of electronic monitoring, which can increase the number of monitored actions and remove the observation bias.^21^

We developed a novel wearable electronic device that monitors and provides real-time, personalized feedback to HCWs based on a simplified and customized HH action. This device is the result of an investigator-initiated partnership between 3 Swiss institutions: the University of Geneva Hospitals (HUG) and Faculty of Medicine, the Haute école du paysage, d’ingénierie et d’architecture de Genève (HEPIA), and iQati, a start-up medical device company.

We aimed to test the impact of the electronic device on compliance with the WHO “5 Moments” for HH and on the quality of hand rubbing in daily patient-care activities. We hypothesized that the use of the device would create a permanent sense of being observed on the HCWs, the so-called positive Hawthorne effect,^22^ that is known to influence compliance with HH. We also hypothesized that the use of the device would directly increase the quality of HH action reflected by the volume of ABHR used and the duration of HH.

## Methods

### Study Design

This study was a Swiss National Research Foundation–funded, investigator-initiated, single-center (ie, HUG) project. We conducted a stepped-wedge, cluster randomized, and controlled open-label trial. This study design was preferred to a classic parallel cluster randomized trial because the device was assumed to be a quality improvement tool to which all participating HCWs should have access.

The trial was approved by the Regional Research Ethics Committee, and all participating HCWs provided written informed consent. This study followed Consolidated Standards of Reporting Trials (CONSORT) reporting guideline. A full trial protocol is available in Supplement 1.

### Participants

All wards of the geriatric hospital at HUG, including outpatient clinics and the emergency department, were eligible to participate if at least 5 permanent HCWs volunteered. HCWs were not eligible if, during the study period, they: (1) planned to leave the ward; (2) worked in more than 1 ward (risk of contamination bias); (3) had more than 3 consecutive weeks of vacations scheduled for the study period; or (4) used an ABHR agent other than the standard at HUG due to skin allergy. HCWs were recruited over the course of 26 10-minute information sessions at clinical nursing and medical staff meetings, as well as by posters and leaflets (eFigure 1 in Supplement 2).

### Settings

HUG is a 1900-bed, tertiary care university hospital with approximately 50 000 admissions per year. The geriatric hospital is a 300-bed, freestanding building, located at a separate site in Geneva, with approximately 10 000 admissions per year.

### Intervention

The intervention consisted of providing real-time individual feedback to HCWs after 15 seconds of hand rubbing and the application of a hand-sized volume of ABHR. Feedback was provided by the novel electronic wearable device (SmartRub), which consists of 2 elements—a bottle and a wristband (eFigure 2 in Supplement 2). Each individual ABHR bottle that is widely used at HUG was equipped, during the transition and intervention periods, with a volumetric flow meter. The flow meter measured the volume of ABHR poured onto hands and during the intervention period it also provided feedback by vibrating as soon as the predefined volume had been applied. The volume was determined for each HCW by taking into account the surface area of each individual’s hands,^23^ as described in previous studies.^9,12,18^ The wristband, made from silicone, was worn during the transition and intervention periods. It measured the duration of each HH action, and during the intervention period it also vibrated after 15 seconds independently of the hand rubbing duration performed by the HCW.^11,12^

Instructions regarding the duration of hand rubbing and the volume of ABHR were solely delivered by the vibration of the electronic device during the intervention period. No additional educational sessions were organized during the study.

HCWs were asked to place the device into a charging station at the end of each shift and to collect it upon starting the subsequent shift. The device recorded the date and time of use, volume of applied ABHR, duration of hand rubbing, and whether feedback was provided to the HCW or not for all of the HH actions. Overall sensitivity and specificity of this novel electronic wearable device (to correctly identify a HH action) were 94.1% (95% CI, 91.4%-96.2%) and 99.0% (95% CI, 97.5%-99.7%), respectively.^24^

### Study Periods and Randomization

Study duration, including baseline, transition, and intervention periods was approximately 6 months, followed by a 2-month washout and a 1-month follow-up period. HH observations were performed at least once a month throughout the baseline, transition, intervention, and follow-up periods.

At baseline period, HCWs did not wear the devices. During the transition period, the novel electronic wearable device was worn but the feedback mode (vibration) was not activated, although the device actively monitored the volume of ABHR and duration of hand friction for each HH action. During the intervention period, the feedback mode of the device was activated and the monitoring of practices by the device continued. At follow-up, HCWs did not use the device.

After an initial common 1-month baseline period, 3 wards per month were randomly assigned to start with the 1-month transition period, followed by the intervention period. The length of the baseline and intervention periods were thus inversely related and varied from 1 to 4 months according to the group of randomization (eFigure 3 in Supplement 2).

In total, 12 wards were randomized following a computer-generated block randomization (1:1:1:1) performed by an independent statistician. Numbered opaque envelopes allowed allocation concealment.

Wards were informed the day before shifting from baseline to transition period. Because of the nature of the study, it was not possible to mask study participants or observers after the baseline period.

### Primary Outcome

The primary outcome was overall HH compliance, as monitored by direct observation of HH and applying the WHO guidelines.^5^ HH opportunities were identified, and rubbing or washing actions were recorded.^25^ HH compliance was calculated as a proportion of HH actions divided by the identified HH opportunities. Three validated observers performed HH observations during audit sessions on weekdays and day shifts. Each HCW was observed at least once per month, with each observation period consisting of a minimum of 6 HH opportunities. HH compliance at the individual HCW level (closed cohort) between intervention and baseline was then compared.

### Secondary Outcomes

Secondary outcomes were: (1) volume of ABHR and duration of hand rubbing for each HH action in transition and intervention periods, as automatically recorded by the device; (2) adherence to device use (ie, hours of device use per day) and frequency of HH (HH events per hour), as automatically recorded by the device; (3) HH compliance of participants during follow-up by direct HH observation; (4) ABHR consumption per ward and study period, as per pharmacy dispensing and device data; (5) adverse events spontaneously reported to the study team by HCWs; and (6) satisfaction and perception of device usefulness assessed by mean of a poststudy questionnaire and focus group discussions.

### Statistical Analysis

Sample size was estimated by hypothesizing that wearing the device would improve HH compliance by a relative 20%, going from 69% (the 2015 HH compliance at HUG) in baseline to 83% in the intervention period. This corresponds to a 0.35 standardized difference in proportions.^26^ We used an intraclass correlation coefficient of 0.015 (based on the 2013-2014 HH compliance data at HUG), leading to a design effect of 1.06.^27^ Considering a study power of 80%, and an α error fixed at 5% (2-sided), we estimated that 12 wards (3 per group, 4 groups) with at least 5 HCWs per ward would be needed to test our study hypothesis.^28,29^ Continuous variables were reported by means (with SDs or 95% CIs) or medians (interquartile ranges [IQR]) where appropriate. Categorical variables were reported by numbers with relative frequency.

All analyses were conducted on an intention-to-treat (ITT) and a per-protocol (PP) basis, the latter excluding HCWs that did not have at least 1 HH observation in each study period or did not complete the intervention period. Pooled HH compliance between the intervention and the baseline periods was modeled using mixed-effects logistic regression. The effect of length of active device use on HH compliance was assessed by testing for interaction between the randomization group and the study period (baseline vs intervention). The clustering of data was taken into account by including nested random effects for HH opportunities within each audit session, HCWs within each ward, and wards within each group (ie, the hierarchical levels of clustering within the trial were in order of groups, wards, HCWs, sessions, and opportunities).

All analyses were adjusted for covariates comprising HCWs’ demographic characteristics (ie, age, sex, profession, years of work experience, years since last HH training, full-/part-time work), ward specialty (internal medicine, geriatrics, emergency department, ambulatory care), workload (mean HH opportunities per minute) and the type of ABHR used (gel, rinse). The models also included time of exposure to the study (number of days between the start of the study and the individual HH session).

We compared separately the volume of applied ABHR and the duration of hand rubbing between the transition and intervention periods by performing a mixed linear regression model (with each outcome used as dependent variables). We assessed the effect of length of active device use on volume and duration of hand rubbing by testing for interaction between the randomization group and the study period (transition vs intervention). Analysis used HCW-level data clustered within the ward level and group level (ie, hierarchical levels of nested random effects, in order: groups, wards, and HCWs). All analyses were adjusted for the same covariates as for the primary analysis. In addition, we calculated the proportion of correct HH actions, which were defined as a sufficient volume ABHR applied (as per hand size) and hand rubbing for at least 15 seconds. Finally, we calculated the HH compliance of all HCWs and per group for the follow-up period.

Two-sided *P* values < .05 were considered statically significant. All analyses were performed using Stata IC version 16.0 (StataCorp).

## Results

**Figure 1.**
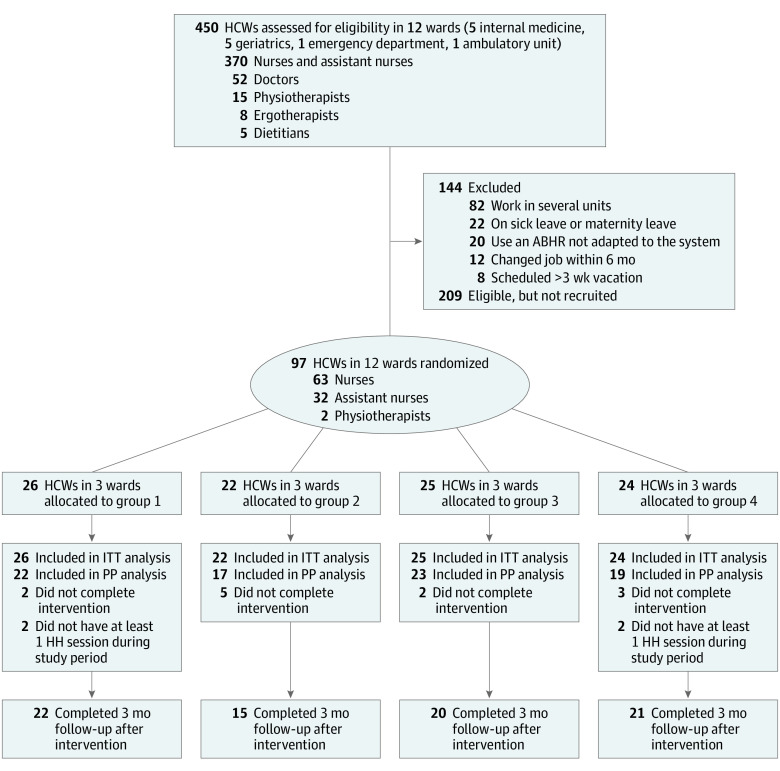
Flowchart of the Study ABHR indicates alcohol-based handrub; HCWs, health care workers; ITT, intention-to-treat; PP, per protocol.

All 12 wards of the geriatric hospital at HUG were eligible and were recruited from June 1 to 30, 2017. There were 4 medical wards, 6 geriatric wards, 1 ambulatory unit, and the emergency department included. Of the 306 eligible HCWs, 97 volunteered and were included in the study (Figure 1). There were 80 women participants, and the median (IQR) age was 42 years (33-53); 63 were nurses, 32 were assistant nurses, and 2 were physiotherapists. Baseline characteristics of wards and HCWs per group are presented in Table 1. See eFigure 3 in Supplement 2 for a summary of the dates of the study periods.

**Table 1.** Characteristics of Wards and Health Care Workers (HCWs) by Group at Baseline

Characteristics	No. (%)			
	Group 1 (4-mo intervention)	Group 2 (3-mo intervention)	Group 3 (2-mo intervention)	Group 4 (1-mo intervention)
Wards	3	3	3	3
Type of ward				
Ambulatory	1 (33.3)	0	0	0
Geriatric	1 (33.3)	2 (66.7)	1 (33.3)	2 (66.7)
Medical	1 (33.3)	1 (33.3)	1 (33.3)	1 (33.3)
Emergency	0	0	1 (33.3)	0
Inpatient beds	57	93	59	80
HCWs working in patient care	77	107	93	93
Participating HCWs	26 (33.8)	22 (20.6)	25 (26.9)	24 (25.8)
Women	19 (73.1)	18 (81.8)	23 (92.0)	20 (83.3)
Age, y				
Mean (SD)	41.6 (11.2)	44.6 (9.2)	40.2 (12.6)	41.9 (12.2)
Median (IQR)	41.5 (35-52)	45 (36-53)	39 (27-52)	43 (30.5-53)
Professional categories				
Nurses	19 (73.1)	12 (54.5)	14 (56.0)	18 (75.0)
Auxiliary Nurses	7 (26.9)	9 (40.9)	10 (40.0)	6 (25.0)
Physiotherapists	0	1 (4.6)	1 (4.0)	0
Part-time work	12 (46.1)	14 (63.6)	15 (62.5)	14 (58.3)
Experience duration, y				
Mean (SD)	17.2 (11.9)	20.2 (10.7)	13.6 (11.4)	17.9 (12.5)
Median (IQR)	18 (8-28)	20 (13-30)	11.5 (2.5-25)	22 (3-26)
Delay since last HH training, y				
Mean (SD)	3.0 (4.2)	3.9 (5.2)	2.2 (2.5)	5.8 (5.4)
Median (IQR)	1 (0-2)	1 (1-5)	2 (1-2.5)	3 (1-10)
Category of hand size^a^				
Small	11 (42.3)	11 (50.0)	19 (76.0)	13 (54.2)
Medium	6 (23.1)	6 (27.3)	5 (20.0)	5 (20.8)
Large	9 (34.6)	5 (22.7)	1 (4.0)	6 (25.0)
Type of ABHR				
Rinse	16 (61.5)	11 (50.0)	11 (44.0)	17 (70.8)
Gel	10 (38.5)	11 (50.0)	14 (56.0)	7 (29.2)

Abbreviations: ABHR, alcohol-based handrub; HH, hand hygiene; IQR, interquartile range.

^a^ Category of hand size was defined in Bellissimo-Rodrigues et al.^18^

Overall, 750 HH observation sessions were conducted, resulting in 6878 HH opportunities. The median (IQR) duration of each HH session was 18 (15-23) minutes. We observed a median of 72 (61-84) HH opportunities per HCW during a median of 8 (7-9) observation sessions.

**Figure 2.**
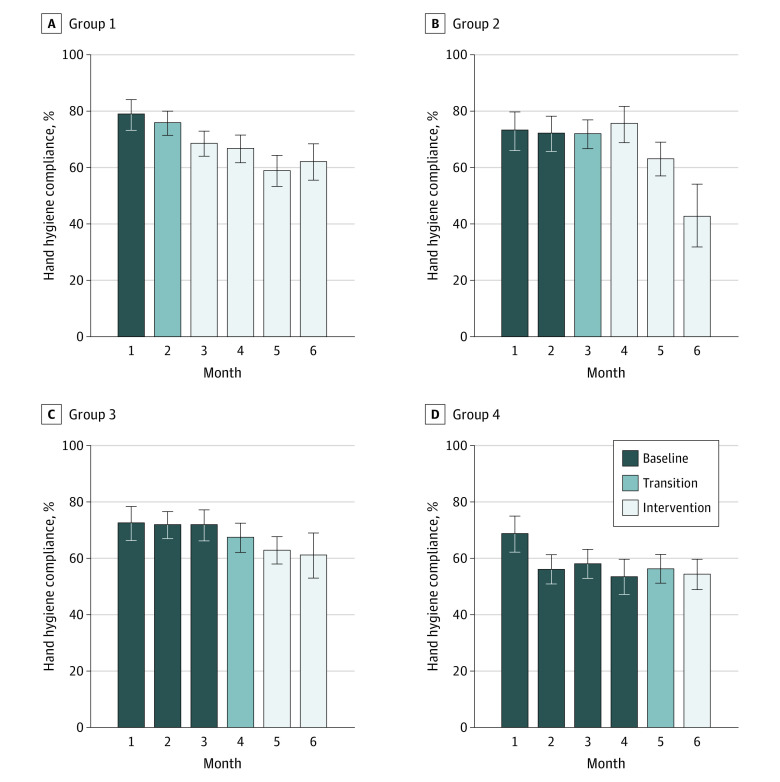
HH Compliance by Periods, Months, and Group

HH compliance rates during baseline, transition, and intervention periods were 66.6% (95% CI, 64.8%-68.4%), 67.9% (95% CI, 65.4%-70.3%), and 62.9% (95% CI, 61.1%-64.7%), respectively. Compliance by group and study period and month are summarized in Table 2 and Figure 2.

**Table 2.** Length of the Study, Number of HH Opportunities, Actions Observed, and HH Compliance in Total, by Group and Period

Characteristic	Group 1 (4-mo intervention)	Group 2 (3-mo intervention)	Group 3 (2-mo intervention)	Group 4 (1-mo intervention)	Total
No. of study days					
Baseline	32	68	118	160	378
Transition	36	50	42	29	157
Intervention	146	96	60	31	333
Compliance with HH, % (95% CI)	68.6 (66.5-70.6)	68.9 (66.2-71.5)	68.2 (65.9-70.4)	57.3 (55.1-59.5)	65.4 (64.2-66.5)
Compliance with HH per period, opportunities/actions					
Baseline					
Total	181/229	277/381	612/848	707/1210	1777/2668
% (95% CI)	79.0 (73.2-84.1)	72.7 (67.9-77.1)	72.2 (69.0-75.2)	58.4 (55.6-61.2)	66.6 (64.8-68.4)
Transition					
Total	299/394	229/318	224/332	213/378	965/1422
% (95% CI)	75.9 (71.4-80.0)	72.0 (66.7-76.9)	67.5 (62.1-72.5)	56.3 (51.2-61.4)	67.9 (65.4-70.3)
Intervention					
Total	882/1363	341/530	334/551	187/344	1754/2788
% (95% CI)	64.7 (62.1-67.2)	64.3 (60.1-68.4)	62.4 (58.2-66.5)	54.4 (48.9-59.7)	62.9 (61.1-64.7)

Abbreviation: HH, hand hygiene.

Crude analysis reveals that, overall, HH compliance at the intervention period was lower than at baseline (OR, 0.62; 95% CI, 0.53-0.72; *P* < .001). After adjusting for covariates, HH compliance was not different between baseline and intervention periods (OR, 1.03; 95% CI, 0.75-1.42; *P* = .85) in the ITT analysis. The randomization group, which took the different lengths of intervention into account, was not associated with a change in HH compliance. Thus, the interaction term was not included in the final model. However, HH compliance in all periods combined was significantly higher in groups 1, 2, and 3 than in group 4 (eg, group 1 vs group 4: 68.6%; 95% CI, 66.5%-70.6% vs 57.3%; 95% CI, 55.1%-59.5%; *P* = .02). HH compliance was inversely correlated with study duration (ie, study days before individual HH observation) (OR, 0.997; 95% CI, 0.994-0.998; *P* < .001), older age (OR, 0.97; 95% CI, 0.95-0.99; *P* = .015), and workload (OR, 0.29; 95% CI, 0.20-0.41; *P* < .001) (Table 3).

**Table 3.** Effect of Real-Time Feedback Provided by SmartRub on Compliance With HH Across Calendar Time and Exposure to the Intervention^a^

Characteristic	OR (95% CI)	*P* value
Baseline	1 [Reference]	[Reference]
Intervention	1.03 (0.75-1.42)	.85
Group 4 (1 mo of intervention)	1 [Reference]	[Reference]
Group 3 (2 mo of intervention)	1.59 (1.14-2.23)	.006
Group 2 (3 mo of intervention)	1.61 (1.15-2.26)	.005
Group 1 (4 mo of intervention)	1.47 (1.03-2.12)	.04
Days since the start of the study until the HH observation	0.997 (0.994-0.998)	<.001
Age at baseline, decrease per mean y of age	0.97 (0.95-0.99)	.02
Men	0.77 (0.57-1.04)	.08
Auxiliary nurse (vs nurses)	1.10 (0.84-1.44)	.49
Experience, y	1.02 (0.99-1.05)	.06
Years since last hand hygiene training	0.99 (0.96-1.02)	.48
Medical/emergency ward (vs ambulatory/geriatric)	1.20 (0.82-1.74)	.34
Part-time (vs full-time)	0.81 (0.63-1.02)	.08
Gel (vs rinse)	1.07 (0.84-1.35)	.59
Workload (mean opportunities per minute)	0.29 (0.20-0.41)	<.001

Abbreviations: HH, hand hygiene; OR, odds ratio.

^a^ Mixed logistic regression model with nested random effect, in hierarchical order of group, ward, health care worker, and session.

In the per-protocol analysis, 81 (83.5%) HCWs were analyzed: 22 (91.7%) in group 1, 17 (77.3%) in group 2, 23 (92%) in group 3, and 19 (79.2%) in group 4. The fitted model confirmed that overall HH compliance was not significantly different between intervention and baseline periods (OR, 0.99; 95% CI, 0.71-1.40; *P* = .99).

Ninety-three of the included 97 HCWs (95.9%) wore the novel electronic wearable device. The bottles and bracelets recorded 65 818 and 45 813 actions in total (eTable in Supplement 2).

The individually applied ABHR volume increased during the intervention period (feedback on) (median [IQR], 1.71 [1.01-2.76] mL) as compared with the transition period (feedback off) (1.12 [0.76-1.68] mL). Correct HH actions, as per correct application of volume of ABHR only, increased from 10.2% (95% CI, 9.8%-10.6%) during transition to 30.5% (95% CI, 30.0%-30.9%) during intervention (eTable in Supplement 2). After adjusting for covariates, the effect of the intervention on higher applied ABHR volume was significant in every group.

Notably, the duration of hand rubbing also increased during intervention (median [IQR], 8 [4.5-15.5] seconds as compared with the transition period, 6.5 [4.5-10.5] seconds). Correct HH actions, as per correct duration only, increased from 11.4% (95% CI, 10.6%-11.9%) in transition to 26.9% (95% CI, 26.4%-27.4%) (eTable in Supplement 2) during intervention. After adjusting for covariates, the effect of the device on the duration of hand rubbing was significant in every group.

Follow-up HH observations at 3 months after the end of the intervention (March 2018) were performed on 78 (80.4%) HCWs. A total of 790 opportunities were observed. The overall HH compliance was 63.4% (95% CI, 60.1%-66.8%). Compliance at follow-up was not different from baseline (66.6%; 95% CI, 64.8%-68.4%; *P* = .09) or intervention periods (62.9%; 95% CI, 61.1%-64.7%; *P* = .79). Compliance at follow-up per group was: 63.6% (95% CI, 57.2%-69.9%) in group 1, 63.2% (95% CI, 55.0%-71.4%) in group 2, 63.9% (95% CI, 57.6%-70.2%) in group 3, and 62.9% (95% CI, 56.2%-69.6%) in group 4 (*P* = .99).

The total participants experiencing any adverse event was 19. All events were skin related: 6 reports of dry skin due to changing from gel to rinse (which subsided after changing back to gel), 3 reports of itching from the bracelet (which subsided after temporarily stopping bracelet use), and 9 reports of dry skin due to using more ABHR than previously. These adverse events directly motivated 2 dropouts (1 related to bracelet itching and 1 to dry skin). As judged by investigators, no serious adverse event was reported.

Device satisfaction and perception were evaluated by questionnaires and focus group discussions. Results are reported elsewhere.^30^

## Discussion

This stepped-wedge cluster randomized clinical trial tested the effect of a novel electronic wearable device that provides feedback on appropriately applied ABHR volume and sufficient duration of hand rubbing, on both compliance and quality of HH practices. Our study showed that wearing the novel electronic wearable device did not improve HH compliance. However, wearing the device did improve the quality of the HH action, with a significant increase in both ABHR volume applied and in the duration of hand rubbing.

Intriguingly, we observed a gradual decline in HH compliance throughout the study, from 73.5% (95% CI, 70.5%-76.5%) during the first month to 56.6% (95% CI, 53.2%-60.1%) during the last month. This time trend was unexpected and, likely, hampered the assessment of the effect of the device on HH compliance. We hypothesize there was a significant Hawthorne effect when HCWs were observed for the first time during the baseline period, which resulted in overestimated HH compliance. The same auditors performed repeated observations of the same HCWs, and HCWs may have become accustomed to them over time. Thus, the presence of the auditors may not have sparked immediate behavior change in subsequent observations. This would result in regression of an initial Hawthorne effect, resulting in HH compliance falling back to routine behavior. This phenomenon of habituation has been previously described in Chen et al.^31^

In contrast, monitoring of HCWs’ behavior with a wearable device might be less affected by those variables. This advantage probably allowed detecting the effect of the device feedback on HH quality. Notably, we observed an absolute increase of around 20% in the correct compliance with both the volume of applied ABHR and on the 15-second duration of hand rubbing. It has been widely shown that these parameters are major determinants of the microbial efficacy of the HH action.^11,12,13,18,19^ However, the clinical significance of these results remains unknown, as our study was not designed to assess the impact of improving the quality of the HH action on the reduction of HAI.

The implementation of electronic monitoring systems raises issues related with transparency and confidentiality,^32^ accuracy, and adaptation to workflow.^33^ We observed good buy-in from HCWs, as the proportion of dropouts related to the use of the device was very small. In a questionnaire performed after the study, the majority (55/70 participants) of HCWs providing feedback agreed that the device was a helpful reminder for correct HH. However, 27% (19/70 participants) were concerned about confidentiality, and 23% (16/70 participants) reported that using the wristband interfered with their clinical activities.^30^ Importantly, the device might not be applicable in settings where a “bare below elbows” strict policy applies. However, we believe that its material (medical silicone), its ergonomics, and the ease of disinfection using any detergent disinfectant might allow for an implementation of the device in these settings for occasional educational interventions. Finally, we have previously tested the device’s sensitivity and specificity in a range of laboratory and clinical conditions.^24^

The most recent systematic review on the effect of monitoring technologies on HH adherence suggested that electronic devices have the potential to change HH behavior.^32^ However, there is a lack of clinical trials with solid designs including system-independent, relevant outcomes.^22,32,34^ We believe that the design of this stepped-wedge, cluster randomized clinical trial contributed to raise the standards of methodological quality of studies on this topic.

Our study is a proof of concept of the benefits of a wearable device on improving the quality of HH in clinical practice and it opens perspectives for new strategies on HH improvement. The great majority of HH monitoring devices focus on dispensing events or proxies for HH indications.^21^ This electronic wearable device is unique in its potential to improve HH by interacting directly with the HCW during HH, in real time and during daily routine.

### Limitations

Our study had several limitations. This was a novel device and, not surprisingly, we faced a series of technical challenges. Problems included random inactivation of the devices (more frequent with the wristband) and loss of data because of errors in data transfer. These prevented us from calculating predetermined secondary outcomes, such as the frequency of HH events, adherence to device use, and ward-level ABHR consumption. The lack of such data did not allow us to analyze if trends of observed HH compliance were different from trends of performed HH events. In addition, the implementation of the study revealed some difficulties. For example, we faced problems related to the production and delivery of the devices that induced delays in the pre-established dates of rollout from baseline to transition in some groups. However, we did manage to respect the stepped-wedge design of the study.

## Conclusions

Wearing a novel electronic wearable device did not improve HH compliance but did lead to an improvement of the quality of HH action as measured by an increase in the volume of ABHR per hand action and in the duration of hand rubbing. The gradual decline in HH compliance may have been unrelated to the use of the device and associated with a high-magnitude Hawthorne effect at baseline. In order to reach its full potential, this innovative device merits further development and adequate testing in future studies.

## Notes

Supplement 1. **Study Protocol**

Supplement 2. **eFigure 1. **Poster Used to Recruit Participants to the Clinical Trial Aiming to Test a Novel Electronic Wearable Device (SmartRub) **eFigure 2. **Photograph of SmartRub Device **eFigure 3. **Starting Dates and Length of Periods of the SmartRub Study (Excluding Wash-out and Follow-up Periods) **eTable. **HH Practices: Description of Number of Actions, Volume of Applied Alcohol-Based Handrub and Duration of Hand Rubbing, as Well as Overall Correctness of Each Action Per Group and Period as Recorded by SmartRub

Supplement 3. **Data Sharing Statement**
