# Supplementary material for: Effect of Wearing a Novel Electronic Wearable Device on Hand Hygiene Compliance Among Health Care Workers: A Stepped-Wedge Cluster Randomized Clinical Trial
Source: JAMA Netw Open. 2021 Feb 8;4(2):e2035331. doi: 10.1001/jamanetworkopen.2020.35331 (PMC7871189; doi:10.1001/jamanetworkopen.2020.35331)
Supplement: Supplement 1. — Study Protocol [file jamanetwopen-e2035331-s001.pdf]

Impact of using a device providing individual feedback on healthcare workers hand hygiene behaviour: a stepped wedge cluster-randomized clinical trial

SmartRub®

Clinical Study Protocol

A stepped wedge cluster-randomized clinical trial assessing the impact of SMART-RUB® on hand hygiene compliance

Study Type:	Clinical trial<br>Improvement of clinical care quality
Study Categorisation:	Risk category A
Study Registration:	ISRCTN25430066 , 22/05/2017 http://www.isrctn.com/ISRCTN25430066
Study Identifier:	None
Principal Investigator:	Didier Pittet
Investigational Product:	Device that measures the quantity of alcohol-based handrub and the duration of hand friction in each hand hygiene action
Protocol Version and Date:	Version 2.3, 02/05/2017

Signature Page

Study Title	Impact of using a device providing individual feedback on healthcare workers hand hygiene behaviour: a stepped wedge cluster-randomized clinical trial

The Sponsor-Investigator and trial statistician have approved the protocol version [2.3 (dated 02.05.2017)], and confirm hereby to conduct the study according to the protocol, current version of the World Medical Association Declaration of Helsinki, ICH-GCP guidelines or ISO 14155 norm if applicable and the local legally applicable requirements.

Sponsor-Investigator: Didier Pittet

Place/Date Signature

Table of Contents

1. ABBREVIATIONS 4
2. SYNOPSIS 5
3. LAY SUMMARY 10
4. BACKGROUND 11
5. STUDY RATIONALE 15
6. AIMS AND HYPOTHESIS 15
7. STUDY DESIGN 16
8. SAMPLE SIZE 18
9. DATA ANALYSIS 19
10. RANDOMIZATION AND BLINDING 19
11. SETTING 20
12. STUDY ACTIVITIES 21
13. DATA COLLECTION 28
14. PRIMARY AND SECONDARY ENDPOINTS 33
15. STUDY POPULATION & ELIGIBILITY CRITERIA 35
16. INTERVENTION: THE ELECTRONIC DEVICE 35
17. SAFETY 37
18. WITHDRAWAL PROCESS 37
19. DATA MANAGEMENT 37
20. MONITORING 38
21. CRITERIA FOR ENDING OF TRIAL / DEFINITION OF END OF TRIAL 38
22. ETHICAL CONSIDERATIONS 38
23. TIMEFRAME 38
24. LIMITATIONS 38
25. IMPORTANCE AND IMPACT 39
26. REFERENCES 40
27. APPENDICE 1 47

1. Abbreviations

ABHR: alcohol-based hand rubs

CDC: Centers for Disease Control and Prevention

CRF: case report form

ECDC: European Centre for Disease Prevention and Control

HAI: healthcare-associated infections

HCW: healthcare workers

HH: hand hygiene

HN: head nurse

HUG: University Hospitals of Geneva

WHO: World Health Organization

2. Synopsis

**Sponsor/Sponsor-Investigator**	Didier Pittet
**Study Title:**	Impact of using a device providing individual feedback on healthcare workers hand hygiene behaviour: a stepped wedge cluster-randomized clinical trial
**Short Title/Study ID:**	SmartRub®
**Protocol Version and Date:**	Version 2.3 of the 02.05.2017
**Trial Registration:**	Trial registered in ISRCTN25430066, 22/05/2017<br>http://www.isrctn.com/ISRCTN25430066
**Study Category with Rationale**	Risk category A<br>This is a clinical trial of quality improvement of healthcare. Its objective is to improve compliance of healthcare workers with hand hygiene.
**Phase of development**	Not applicable.
**Background and Rationale:**	Healthcare-associated infections (HAI) are a major public health problem, with an estimated hundreds of millions of new episodes occurring annually worldwide. They affect approximately 7% and 10% of all hospitalized patients in developed and developing countries, respectively, and are responsible for millions of deaths worldwide each year.<br>It is well recognized that as much as 50-70% of the HAI infections episodes are transmitted or inoculated by healthcare workers (HCWs)' hands due to the lack of proper hand hygiene (HH), which remains the most efficient method to prevent its occurrence. For this reason, the World Health Organization (WHO) recommends a Multimodal Strategy with 5 elements to improve HH practices in the healthcare setting. In that list, recommendation number 3 (performing observation of HH practices and providing timely performance feedback) is one of the most challenging because evaluating HH practices by direct observation is a time-consuming and costly task.<br>An electronic device intended to continuously monitor HH practices and to provide a real-time feedback to healthcare workers could be very useful. The device intended to be studied in this study consists in a bracelet and a clip added to the individual bottle of ABHR that measures and provides feedback to the HCW on the volume of ABHR and duration of hand friction of each individual hand hygiene action performed. The volume of ABHR and duration of hand friction are considered surrogate markers of hand hygiene gesture quality. This device has potential advantages over traditional strategies to promote HH: it is not expensive and it is simple to implement; it may provide a continuous sense of "being observed" to HCWs and could influence their behaviour regarding HH, potentially improving compliance when they are not observed; it may also influence the quality of HH action. Besides, hand hygiene quality is important and it is rarely assessed in hand

	hygiene monitoring. We have performed a series of experimental-based studies to address the hand hygiene action quality and we have established an optimized volume of ABHR, according to the hand size of the HCW, and duration of hand friction of 15 seconds. These parameters will be applied in the current study.
**Objective(s):**	We aim to identify the effectiveness of using a new device providing automatic, immediate and personal feedback regarding the volume of ABHR and duration of hand friction during each hand hygiene gesture in promoting HH compliance amongst HCWs performing patient-care activities, as well as in enhancing the quality of the HH action.<br>We hypothesize that compliance with HH amongst HCWs may be improved by at least a relative 20%, from baseline to intervention, if they receive a continuous feedback about the quality of their hand hygiene gesture during daily patient care when using the hand hygiene device.
**Outcome:**<br>**Primary Outcome**<br>**Secondary Outcome**	Primary outcome:<br>Hand hygiene compliance is measured by direct observation by well-trained IPC professionals according to the WHO methodology at the individual HCW level at six time-points (once a month) in the three study periods (baseline, transition period and intervention).<br>Secondary outcomes:<br>1. Hand hygiene quality is assessed using volume of ABHR poured by HCWs and duration of handrubbing in each HH action using the device collecting automatic and continuous data<br>2. Frequency of hand hygiene is measured using the device collecting automatic and continuous data<br>3. Adherence to hand hygiene device is measured using how many hours the device is used by HCWS using the device collecting automatic and continuous data<br>4. Hand hygiene compliance at follow-up is measured by direct observation by well-trained IPC professionals to assess for the sustainability of the intervention at three month follow up<br>5. Hand hygiene compliance and alcohol-based handrub consumption at a unit level is measured using the HH compliance data is recorded on a regular basis by the IPC professionals and ABHR consumption is provided monthly by the pharmacy<br>6. Satisfaction and perception of usefulness of the device by HCWs is measured using a questionnaire distributed to participants and some focus group discussions with HCWs participating in the study to evaluate their experience with the device use at the end of the intervention<br>7. Hand hygiene quality and HH compliance among HCWs working in several units during the study period. This is done as a sub-study with HCWs that are willing to participate but that are excluded from the main study due to working in several units (meaning that they can't be allocated to a cluster). This group of HCWs receives the device only in the fifth month of the study, when all the units have already started the study intervention, to avoid contamination.These HCWs data does not contribute to the primary outcome analysis.<br>8. Adverse events related to the device are measured using a list of open responses (ie., skin irritation, injury to patient, etc) asked to participants after each HH session

	9. Bloodstream infections (BSI) surveillance are measured using the data routinely and prospectively collected by the IPC program in order to analyse if there is a change in incidence of BSI in the units during the study period
**Study Design:**	Stepped wedge, cluster-randomized, controlled, open-label clinical trial. The hospital ward is the unit of randomization. Primary outcome will be assessed at the HCW level (closed cohort).
**Inclusion/Exclusion Criteria:**	Study population: healthcare workers working at the University Hospitals of Geneva (HUG) with patient care activities.<br>All **units** will be screened to participate in the study.<br>Inclusion criteria: All units are eligible to participate in the study.<br>Exclusion criteria: Units without patient-care activities.<br>All **HCWs** in eligible wards can participate in the study.<br>Inclusion criteria: All HCWs working in patient-care activities.<br>Exclusion criteria:<br>1. Work or will work in several different units during the six months after study start<br>2. Who will leave the unit in the six months after study start<br>3. Who have more than three consecutive weeks of vacations in the six months after the study start<br>4. Who don't use the standard ABHR at HUG
**Measurements and Procedures:**	This will be a stepped-wedged, cluster randomized, controlled, open-label, clinical trial in the University Hospitals of Geneva (HUG). The intervention will consist of wearing an electronic device intended to provide immediate, individualized feedback to HCWs at the point of each HH action. Feedback is provided regarding the quality of his/her action in terms of duration and volume of ABHR used, both parameters reflecting the quality of HH. In addition, the device will also record information regarding the date, hour, volume of ABHR, and duration of each HH action performed by the HCW using it.<br>A pragmatic stepped wedge design where clusters (wards) will be rolled out randomly and sequentially from no intervention (baseline), to inactive device (transition period) and finally to active device (intervention) followed by a period of (at distance) follow-up was designed.<br>Statistical analyses will take into account some clustering in the data at the ward level and generalized linear mixed models with a random effect on the intercept at ward level will be performed. Random block randomization of wards will be done. Hand hygiene observers will be blinded regarding allocation of wards until the device delivery to the wards.

**Study Intervention and comparators:**	Participants are given an electronic device in the form of a wrist band, and a pocket-size individual bottle of alcohol based hand rubs (ABHR) with a "clip" inside that provides a personal, automatic and individualised feedback on hand hygiene quality surrogate markers to HCWs. This device was developed at the University of Geneva Hospitals and Faculty of Medicine in cooperation with the School of Engineers of Geneva. In the designed stepped wedge study, the clusters (units) are randomly and sequentially rolled out from baseline, to a fixed transition period of one month and followed by the intervention period. The length of the time-points is one month and the study has 4 steps.<br>Random block randomisation of units is done. Hand hygiene observers and participants are blinded regarding allocation of units until the device delivery.<br>Step 1: This step consists of a baseline period of one month, a transition period of one month and the intervention period of four months.<br>Step 2: This step consists of a baseline period of two months, a transition period of one month and the intervention period of three months.<br>Step 3: This step consists of a baseline period of three months, a transition period of one month and the intervention period of two months.<br>Step 4: This step consists of a baseline period of four months, a transition period of one month and the intervention period of one month.<br>The baseline period there is no intervention, as it corresponds to standard of practices (therefore no device is in use). In the transition period HCWs use the device, but they do not receive any feedback about their correct practices. In the intervention period HCWs use the device that provides them with feedback about how well they are doing with their hygiene compliance.<br>Throughout all the study periods HCWs perform their daily activities and are observed regarding their compliance with hand hygiene. This study will be conducted at the Geriatric Hospital, one of the 8 sites of HUG. In 2018, we aim to perform a larger study to test the device in the acute care hospital sites of HUG.
**Number of Participants:**	In total, 60 participants (5 HCW per ward, 12 wards) would be needed to answer the research question according to the sample size calculation.
**Study Duration:**	The protocol will be implemented from May 2017 until February 2018. The data entry will be performed at the same time. We will perform the data cleaning and analyze the data from February 2018 to April 2018. After that we will present the results on national and international conferences, as well as write a manuscript for submission.
**Study Schedule:**	From May 2017.
**Investigator(s):**	Didier Pittet<br>Yves Martin<br>Daniela Pires<br>Angèle Gayet-Ageron<br>Walter Zingg<br>Carolina Fankhauser<br>Ermira Tartari<br>Josiane Sztajzel-Boissard

	Fernando Bellissimo-Rodrigues<br>University Hospitals of Geneva, Infection Prevention and Control Programme, Rue Gabrielle-Perret-Gentil 4, CH-1211 Genève 14
**Study Centre(s):**	Single-centre.
**Statistical Analysis incl. Power Analysis**	We hypothesized that using the active device will increase the mean compliance with HH by a 20% relative increase from 69% to 83%, corresponding to a standardized difference in proportions of 0.35. Due to the clustered study design, we will apply a correction for the correlation of compliance within the same wards by calculating a design effect. We anticipate recruiting a maximum of 5 HCWs per ward, and will use an intra-class correlation (ICC) coefficient of 0.015 (based on data from the 2013-2014 survey about HH compliance in our institution), leading to a design effect of 1.06. Considering a study power of 80%, and an alpha error fixed at 5%, we would need 12 wards with 5 HCWs included in each one to test our study hypothesis.<br>During a session of 20 minutes of observation by a HCW, we expect to an average of 5 HH opportunities. As each HCW will be observed for 6 sessions, the average number of opportunities per HCW will be 30. For the same HCW, we specifically monitor their compliance with each of the 5 moments for HH. Considering 12 wards to be included, 5 HCWs to be observed within each unit, for 6 sessions of observations for each HCW, the total number of observation sessions will be therefore 360.
**GCP Statement:**	This study will be conducted in compliance with the protocol, the current version of the Declaration of Helsinki, the ICH-GCP or ISO EN 14155 (as far as applicable) as well as all national legal and regulatory requirements.

3. Lay summary

Healthcare-associated infections (HAI) are a major public health problem. They are estimated to represent hundreds of millions of new episodes each year leading to significant mortality and financial losses for health systems. HAI affect approximately 7% and 10% of all hospitalized patients in developed and developing countries respectively, and are responsible for millions of deaths worldwide each year. In parallel, studies have shown that as much as 50-70% of all HAI are transmitted through the hands of healthcare workers (HCWs) due to a lack of proper hand hygiene (HH). As a consequence, HH remains the most efficient method to prevent the occurrence of such infections.

HH seems to be a very simple action but it is insufficiently performed by HCWs in hospitals. The objective of the current study is to improve both the compliance with hand hygiene and the quality of HH action. We would like to assess the role of an electronic device intended to continuously monitor each HH action (volume of ABHR used and duration of hand friction performed by HCWs) and provide real-time feedback. We will conduct a randomized clinical trial with a very robust methodology and a pragmatic approach in several wards at the University Hospitals of Geneva, a large tertiary-care university hospital with long-term successful experience in HH promotion.

Considering that HAI is a major public health problem, we are confident that the device to be tested in our study will contribute to prevent the occurrence of infections and promote institutional safety culture in the future.

4. Background

Burden of healthcare-associated infections

Healthcare-associated infections (HAI) are a major threat to patients in the healthcare setting. They affect approximately 7 and 10% of all hospitalized patients in developed and developing countries, respectively, and are responsible for millions of deaths worldwide each year.[1,2] In Europe, the European Centre for Disease Prevention and Control (ECDC) estimated that during 2011-2012 the prevalence of HAI was 5.7%, which is equivalent to 81,089 patients affected a day.[2] The ECDC also estimated that, in acute-care hospitals alone, 3.2 million patients are affected by HAI each year, directly causing 37,000 deaths, and contributing to 110,000 additional deaths, extra-costs reaching approximately € 7 billion.[2,3] In Switzerland, they cause at least 2,000 deaths corresponding to an annual extra cost of 350 million CHF per year.[4]

Importance of hand hygiene to prevent HAI

It is now well recognized that as much as 50-70% of the HAIs are transmitted or inoculated by healthcare workers (HCWs) hands due to the lack of proper and timely hand hygiene (HH).[5-10] HH may be performed by two different methods, handwashing with soap and water or handrubbing with an alcohol-based handrub (ABHR), the latter being preferred in most clinical situations due to its greater microbiological efficacy, better skin tolerance and more practical application.[6,7,11] In our institution, the University Hospitals of Geneva (HUG), more than 98% of all HH actions are performed using ABHR.

For the last 10 years, The World Health Organization (WHO) has been extensively promoting HH in the healthcare setting to enhance patient safety.[12,13,14] According to the WHO multimodal strategy for HH improvement,[13] five elements are considered essential requirements for achieving best practices, one of which is the monitoring of hand hygiene practices and feedback.

Hand hygiene monitoring

The easiest and less costly way to broadly estimate HH compliance is to regularly measure ABHR and soap consumption.[12] Nonetheless, this measurement will only provide a general assessment of the situation, with no further details on individual compliance for specific indications. Although better than not monitoring anything, this should be used as a single strategy only in low-resource settings, or in low-risk facilities, such as those providing ambulatory care.[15,16] Several more specific approaches have been proposed for HH compliance monitoring. Developed 20 years ago at the HUG, the peer-based direct observation method is still considered the gold standard by WHO.[12,14] This method is able to identify all five main indications for performing HH, which are clinically relevant for both the patient's and the HCW's health,[17] and has been validated and used in a wide range of facilities in different countries,

continents and cultures, shown to be universally efficient.[18-23] Beyond that, it provides both an opportunity for the infection control practitioners to identify local obstacles for HH, and to give immediate customized feedback to the HCW.[24-25] However, the drawback of this gold standard method is that it is time-consuming. Since it does not provide a sustained effect after being implemented, HH observation and correspondent feedback must be continuously promoted, to assure maintenance of high HH compliance.[14,24,25] Another limitation of this method is that it doesn't monitor the quality of the HH action.

More recently, automated electronic monitoring of HH practices have been proposed as an alternative to the direct observation, given that they are likely to consume fewer human resources and provide larger and more representative data sets, and be less subject to observation bias and Hawthorne effect.[26-43] It is now considered a promising tool, opening the possibility of continuously monitoring HH practices and providing feedback to HCWs, eventually enhancing HH practices.[42,43] The major limitations of automated methods are their high costs, and their inability to monitor compliance with HH.[42,43]

One of these novel technologies is video-monitoring direct observation, which has been the focus of some previous studies.[38-41] Its main advantage is that it collects a large amount of data, since it continuously records HH actions and opportunities. However, there is an ethical debate over it, related to patients' privacy. In the other sense, if it focuses upon the room entries and exits it may better preserve patient privacy but will miss a significant portion of HH opportunities.[12,13,17] The video-camera strategy didn't solve the problem of time-consumption related to the direct observation method. Beyond that, costs of implementation may be high, considering the need for multiples cameras split over beds and wards.[38,39] In conclusion, even if promising, this method has several defaults and constraints that minimize its use in routine.[24,30,37] Another technology used for assisting HH observation is the use of counters coupled with ABHR bottles, which can count each time an HH action is taken.[44-58] This is a relatively inexpensive and simple intervention. Combined with HH training and other measures recommended in multimodal strategies, bottle counters did enhance the frequency of HH, when accessed by observational and quasi-experimental studies.[44,49,50,53] However, it may not evaluate HH compliance given that it does not evaluate HH indications. In our opinion, the most promising electronic tools to assist and enhance HH compliance are automated HH monitoring networks.[27-29,32-36,59-74] Based on infrared, radiofrequency, ultrasound, real-time location monitoring, or detectors of alcohol vapors, these systems can detect when an HH opportunity occur, produce a visible or audible sign to remind the HCW to accomplish HH, and record if the action was taken or not. Their main advantages, when compared to the direct observation method, are to continuously monitor HH practices and provide real-time feedback to HCWs, eventually enhancing HH compliance, and consuming less time of the infection control personnel to monitor compliance.[59-74] However, they are based on surrogate markers of some of the main 5 HH indications and not the WHO "5

Moments" concept itself.[17] Cost of implementation and studies based on quasi-experimental design are other limitations of these new technologies.[59-74] Available electronic systems intended to evaluate the quality of the HH action focus on the technique and are based on ultraviolet light (Hand-in-Scan™, HandInScan Ltd., Hungary)[75] and video measurement technology (SureWash™, Glanta Ltd., Ireland).[76,77] They have been successfully used for HH training purposes, but they are not intended to continuously monitor the quality of HH action, which is now thought to be as relevant as the compliance rates.

In conclusion, although promising, to date, no automated monitoring system has proved to be able to monitor and improve HH compliance with all the "5 Moments" as recommended by WHO, nor has been proven effective in preventing HAI, by the use of the gold standard design, randomized controlled trials. [24,30,37]

Hand hygiene action quality: volume of ABHR and duration of hand friction

Great efforts have been made to improve hand hygiene compliance among HCWs worldwide. However, less attention has been devoted to the quality of the hand hygiene action itself, despite this being probably equally important in preventing HAI. Even though compelling evidence shows that inadequate performance of the hand hygiene action can lead to cross-transmission of bacteria,[12,78] it still receives little attention in most healthcare institutions.[79] Contributing factors may include the lack of clear evidence-based guidance on its performance and the absence of tool to conduct monitoring and foster its improvement amongst HCWs.

The World Health Organization (WHO) hand hygiene guidelines[12] address several aspects related to the quality of the hand hygiene action. A specific 6-step technique is recommended in the "*how to handrub*" poster. However, less precise information exists on the volume of ABHR ("palmful") and duration of hand friction (20 to 30 seconds) required to perform an optimal hand hygiene action. The Centers for Disease Control and Prevention (CDC)[80] guidelines for hand hygiene are equally imprecise, mentioning that "*if hands are dry before 10 to 15 seconds, an insufficient amount of ABHR has been used*". Furthermore, the European norm[81] to test hand products also includes 30 seconds of hand friction, but ABHRs can be tested with handrubbing durations of up to 60 seconds to pass the norm without a reference to a volume to be used to pass the norm.[82-84] These heterogeneous and imprecise recommendations reflect the overall poor level of evidence and lack of consensus.

In order to address these controversial issues we have performed several laboratory-based experimental studies at the HUG Infection Prevention and Control laboratory. These studies focused on volume of ABHR and duration of hand friction as surrogate markers of hand hygiene action quality.

In the experimental study focusing on volume of ABHR,[85] we identified that the volume of ABHR used by HCWs directly correlates with the $\log_{10}$ reduction of bacteria in their hands and this is influenced

by their hand size. The bacterial log_{10} reduction was significantly decreased for each supplemental 0.5ml of AHBR (0.28 log_{10}; 95%CI: 0.23 to 0.34, p<0.001) after adjustment on hand size and baseline log_{10} count. The log_{10} reduction was significantly lower for large hands compared to small hands (-1.19 log_{10}; 95%CI: -1.61 to -0.76, p<0.001), and significantly lower for medium hands compared to small hands (-0.57log_{10}; 95%CI: -0.98 to -0.15, p=0.007). As a consequence of those differences, HCWs with large hands achieved a mean reduction of only 1.42 log_{10} ± 1.31, after rubbing their hands with 3mL of ABHR.[85]

We also investigated the influence of handrubbing duration in the reduction of bacterial counts on HCWs hands.[86] We observed that the reduction of bacterial count after handrubbing for 15 or 20 seconds is not significantly different from that achieved after 30 seconds and demonstrated that performing hand friction for 15 seconds is non-inferior to 30 seconds, while controlling for possible confounders. Our results expand and strengthen previous studies' findings.[87,88]

These studies suggest the need for customizing the practice of HH taking into consideration the hand surface area of HCWs, which will indicate the necessary amount of ABHR and the adequate time devoted to each HH action, in order to achieve proper hand antisepsis and, consequently, patient safety. In practice however, we know that HCW perform hand hygiene using low volumes of ABHRs and handrub for short durations. The real duration of handrubbing practiced by HCP in routine care remains largely unknown, but it is certainly less (mean 11.6 seconds [SD ±0.7][88] Additionally, a local evaluation in our facility (unpublished data) indicates that the average volume of ABHR used per handrubbing action was around 1.05 mL and the average duration of friction was 10 seconds.

Development of device to monitor hand hygiene action quality

In this context, our group, in collaboration with the Schools of Engineering and Art and Design of Geneva, developed an electronic device that continuously monitors both the amount of ABHR used and the duration of handrubbing, in each HH action.[89] The device is made of a bracelet to be worn around the wrist by HCWs and a bottle of ABHR with an added "clip" inside. In addition to continuously monitor the frequency of use, the volume of ABHR and the duration of hand friction, it is also able to provide immediate feedback on these two parameters to the individual HCW. We expect this immediate, automatic and personalized feedback on the volume and duration of hand friction to improve the quality of the HH action. Importantly, we expect that with the use of this device the HCWs became more aware of hand hygiene and improve also compliance with the "5 moments".

A pilot study (Appendix 1) was conducted in a ward to test the possible effect of real time feedback on the volume of ABHR used by HCW during patient care. A total of 11 HCW provided care and rub their hands using an ABHR bottle equipped with the monitoring device first (before period)

without feedback and then with feedback during ABHR taking. Overall, the volume of ABHR used increased from a mean (±SD, median; p25-p75) of 1.33 ml (±0.37, 1.33; 1.07-1.54) without feedback to a mean of 3.63 ml (±0.87, 3.60; 3.04-4.11) after feedback. The average duration of handrubbing monitored by the device was 13.5 seconds. The mean (±SD, median, maximal negative error, maximal positive error) of the error is -0.13 sec (±1.43, 0.00, -3.0, 3.2).

5. Study rationale

Available methods for monitoring HH practices are too time and resources demanding. Beyond that, even with the most successful strategies and campaigns, HH compliance reached usually at most 60 to 70%.[20] Thus, there is still room for further improvement in HH compliance. In this sense, the device intended to be studied here has at least three potential advantages over traditional strategies to promote HH: it is not expensive and it is simple to implement; it may provide a continuous sense of "being observed" to HCWs and therefore influence their behaviour regarding HH[90] and it may improve also the quality of HH action, since it provides an immediate feedback to HCW. Besides, hand hygiene quality is important and it is rarely assessed in hand hygiene monitoring. In the last 2 years we have performed a series of experimental-based studies to address the "optimal" hand hygiene action; we concluded that a personalized volume of ABHR (according to the hand size) and a 15 seconds duration of hand friction are needed to an obtain "safe hands". These parameters will be applied in the current study.

6. Aims and Hypothesis

We aim to identify the effectiveness of using a new device providing automatic, immediate and personal feedback regarding the volume of ABHR and duration of hand friction during each hand hygiene gesture in promoting HH compliance amongst HCWs performing patient-care activities, as well as in enhancing the quality of HH action.

We hypothesize that compliance with HH amongst HCWs may be improved by at least a relative 20%, from baseline to intervention, if they receive a continuous feedback about the quality of their hand hygiene gesture during daily patient care when using the hand hygiene device.

7. Study design

We will conduct a **stepped wedge, cluster randomized, controlled, open-label** clinical trial at HUG. The stepped wedge study design was chosen instead of a classic parallel placebo controlled design because stakeholders considered that the device use was a learning opportunity and thus should be made available for all study participants. Furthermore, the study team was limited by the availability of devices and the sequential introduction of those in the wards was appropriate in terms of device production.[91] Data from the pilot studies show that the device is effective in improving the hand hygiene action quality, as measured by the volume of ABHR and duration of hand friction (secondary outcomes; Appendix 1) and no adverse events with its use were registered or are expected.

The **cluster randomization** nature of the study imposed itself due to the frequent interactions of the HCWs in the wards that lead to in-wards behavior cross-contamination.[92] The cluster unit is therefore the ward. Data will be collected from all clusters at all time-points over time.

A stepped wedge trial was designed where clusters (wards) will be randomly and sequentially rolled out from baseline, to a fixed transitory period of inactive device (no feedback, transition period) followed by the intervention period (device with feedback; intervention) (Figure 1). The length of the time-points is one month and the study will have 4 steps. At the beginning of the study none of the wards will be exposed to the intervention (pre-rollout period) and at the end of the study, all wards will be exposed to the intervention (post-rollout period). The pre- and post-rollout periods are fixed at one month. The time of exposure to the active device will be split in 4 steps with a longer period of 4 months in step 1, to a shorter period of exposure of 1 month in step 4 (Figure 1). Thus, the duration of the trial will be 6 months. Additionally, an at distance follow-up period was also designated in order to assess the sustainability over time of the intervention (secondary outcome).

The **primary outcome** will be assessed at the HCW level; repeated measures will be taken from the same HCWs throughout the study in order to assess the change in HH compliance and its relation to the intervention (exposure). It will be thus a closed cohort design. All HCWs will contribute to measures in all time-points of the study (including those in the transition period).

This study will be conducted at the Geriatric Hospital, one of the 8 sites of HUG. In 2018, we aim to perform a larger study to test the device in the acute care hospital sites of HUG.

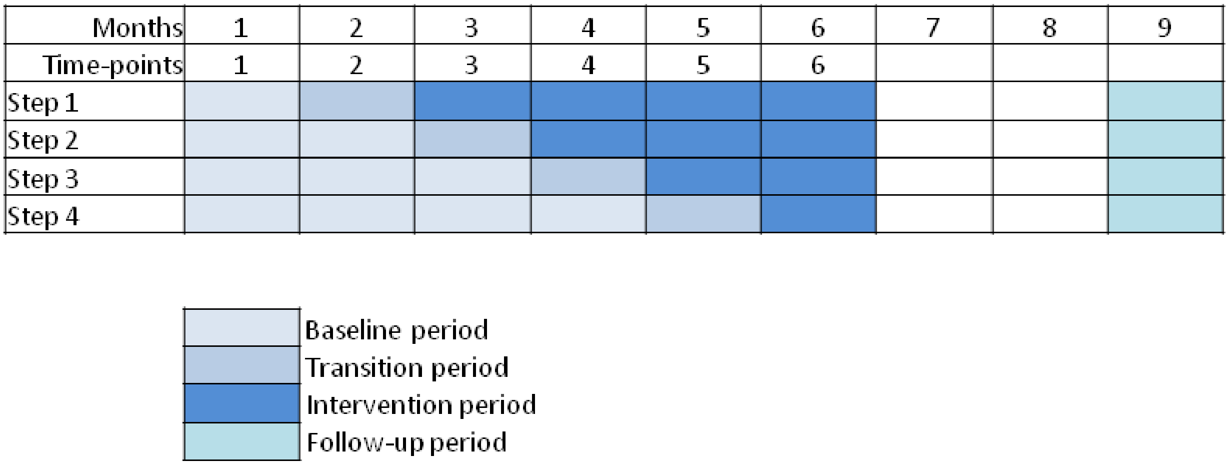

Figure 1: Study design. Stepped-wedge cluster randomized, controlled, open-label clinical trial with 4 steps. Three clusters (or wards) will be allocated to each step.

The study periods (baseline, transition, intervention and follow-up) correspond to:

- *Baseline period*: this period will last from 1 to 4 months, depending on the step to which the ward is allocated. HCWs will perform their normal daily activities and won't have the device. HCWs will be directly observed regarding their HH compliance (primary outcome) once per time-point (monthly).
- *Transition period*: this period will have a fixed duration of 1 month in all steps. HCWs will perform their normal daily activities and will use the device. However, the device won't provide any feedback to the HCW using it. HCWs will be observed regarding their hand hygiene compliance (primary outcome). Data will be also obtained regarding the volume of ABHR used and duration of hand friction continuously by the device (secondary outcome). The device data of this period will be used to adjust for the feedback setting of the device in the intervention period. This period is to assess HCW's compliance with HH after wearing the device that provides no feedback and will obtain baseline data for the secondary outcome hand hygiene quality.
- *Intervention period:* this period will last from 1 to 4 months, depending on the step to which the ward is allocated. HCWs will continue to use the device introduced during the transition period and the device will provide immediate feedback after each hand hygiene action performed by the HCW on the volume of ABHR and duration of hand friction. Data will be collected regarding primary and secondary outcomes.
- *Follow-up period:* this period will have a fixed duration of 1 month in all steps. After the end of the intervention period HCWs will stop wearing the device of the study. Three months after they will be observed regarding their hand hygiene compliance. This period will help evaluate the sustainability of

hand hygiene compliance improvement (secondary outcome) and will not contribute to the primary outcome analysis.

8. Sample size

We hypothesized that using the active device will increase the mean compliance with HH by a 20% relative increase from 69% to 83%, corresponding to a standardized difference in proportions of 0.35.[93] Due to the clustered study design, we will apply a correction for the correlation of compliance within the same wards by calculating a design effect.[94] We anticipate recruiting at least 5 HCWs per ward, and we will use an intra-class correlation (ICC) coefficient of 0.015 (based on data from the 2013-2014 survey about HH compliance in our institution), leading to a design effect of 1.06. Considering a study power of 80%, and an alpha error fixed at 5%, we would need 12 wards and at least 5 HCWs per ward to test our study hypothesis.[95-98]

Table 1: Study power calculations.

	**SW-CRT (with 4 steps)**
Number of time-points/periods	6 (incl. baseline)
Number of HCW per cluster	5
Number of wards per step	3
Total of wards estimated	12
Total number of measures	360
Study power	**0.8219**

In conclusion, with 12 wards and 5 HCWs per ward, we would be sufficiently powered to demonstrate the relative increase of 20% compliance.

During a session of 20 minutes of HH observation by a HCW, we expect to observe an average of 5 opportunities. This value is based on observations made in 2011 at HUG and at pilots performed in preparation for this study. Each HCW will be observed at all time-points of the study (6 time-points) and at follow-up (1 time-point). So, we aim to observe a minimum of 5 HH opportunities for each HCW in each time-point. This corresponds to a minimum of 30 HH opportunities per HCW during the study period- not counting with the follow-up. Considering 12 wards to be included, 5 HCWs to be observed within each unit, for 6 sessions of observations for each HCW, the total number of observation sessions

will be therefore at least 360. For the same HCW, we specifically monitor their compliance with each of the 5 moments for HH.

9. Data analysis

We will present continuous variables using mean±standard deviation (SD) when normally distributed and median±interquartile range otherwise. Categorical variables will be presented by number and relative frequency. Estimation of global HH compliance will be presented with their 95% confidence interval (95% CI) per arm.

All analyses will be done on an intention-to-treat basis. Primary analysis compares the compliance between the intervention (active device) and the control period by use of logistic regression. Analysis will use HCW-level data that will be clustered within the ward level. The unit of clustering will be the ward. We will use the Huber-White sandwich method to calculate robust variance estimates.[99] Time in weeks will be considered in the model in order to assess its effect on the outcome (treated as categorical and continuous variable).

Because we could not completely control for all confounders in a cluster randomized trial design[100] we also plan to adjust the model on individual (sex, professional category), ward (sector of care surgery, rehabilitation or medicine), period of time and fidelity to intervention (device use hours). Unadjusted between-group differences will be presented for completeness.

For the comparison of continuous secondary outcomes (duration of HH action and volume of ABHR used), mixed linear models will be used with HCW-level data clustered within the ward level.

We will perform an as per intention to treat analysis and completed by per protocol analysis.

10. Randomization and blinding

Three randomization lists will be created by a statistician from the Centre de Recherche Clinique at HUG. Random block size randomization will be used to have a balanced number of steps in all the trial. The order of attribution of the steps will be allocated chronologically in sealed envelopes prepared in advance and kept in a confidential place. Once eligibility criteria are verified, the responsible of the trial will open the first envelope and attribute the step at the randomization visit. All HCWs from this participating ward will be enrolled in the randomly attributed step.

Allocation concealment will be guaranteed by the use of opaque envelopes sealed up by an external person who will not participate in the implementation of the study.

Blindness will be applied until the beginning of the transition period for HH observers in charge of assessing the primary outcome, HCWs and head nurse. Due to the nature of the study, it is not feasible to blind the allocation of the ward after the beginning of the transition period (HCWs will need to use the device and HH observers will realize if HCWs are wearing a bracelet or not). The statistician in charge of data analysis will be blinded throughout the trial.

The ward ID will be provided at the randomization visit and will be randomly selected (number from 1 to 12). The HCW ID will also be provided at the randomization visit and will be randomly selected (from 1 to 120). The same and only ID's will be used in the CRFs; only study investigators will have access to the codes.

11. Setting

The study will be conducted at HUG, a tertiary-care university hospital center covering a population of approximately 800,000 inhabitants. It comprises 1,900 beds split into 50 services and provides acute care for approximately 47,000 in-patients per year, equivalent to 286,000 annual hospital-days. The average length of patient stay is 6.4 days. Eight physically independent hospitals constitute this tertiary-care university hospital center. These hospitals are all located in the city of Geneva and provide different types of care. All share the same Infection Prevention and Control (IPC) Programme and similar policies regarding infection control. However, each hospital has different infection control nurses and develops different infection control promotion activities, including hand hygiene activities, according to their needs and motivation. These hospitals are:

- Acute care (Hôpital, bâtiment principal): 23 wards (include Emergency department and Intensive Care units)
- Rehabilitation (Hôpital de Beau-Sejour): 8 wards
- Geriatrics (Hôpital des Trois-Chêne): 12 wards
- Maternity (Maternité): 4 wards
- Paediatrics (Hôpital des enfants): 10 wards
- Psychiatry (Hôpital de psychiatrie): 5 wards
- Palliative care (Hôpital de Bellerive): 7 wards
- Long-term care (Hôpital de Loex): 10 wards

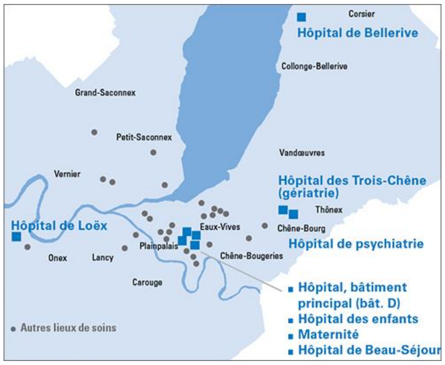

Figure 2: Geographic location of the sectors of the University Hospitals of Geneva (HUG).

12. Study activities

The study was presented to the medical, nurse and healthcare assistant's directors and heads of sectors in December 2016 and their agreement and support was obtained to implement the study in the first semester of 2017.

The study will be conducted at the Geriatric Hospital. All wards will be assessed for eligibility (according to **ward eligibility criteria**) and within wards, HCWs will also be assessed for eligibility (**HCW eligibility criteria**). The eligibility assessment of wards will be performed by the study investigators and assisted by IPC nurses of the HUG. The eligibility criteria of HCWs within eligible wards will be assessed by study investigators with the head nurse and HCWs of each ward at the time of recruitment.

At least 1 month before the study starts a **meeting with the heads** of the wards (nurse and healthcare assistants, physicians, dieticians, physiotherapists) will be held. During this meeting, the detailed study information will be provided.

Pre-randomization study visits will be: **ward baseline visits** to eligible wards, **information visits** to recruit HCWs, **HCW screening and baseline visits** to eligible HCWs and finally a **ward randomization visit** will be performed to enrol HCWs and randomize the ward (see schema in Figure 3).

In each ward the study will proceed from baseline, to transition period, intervention and follow-up. The duration of the baseline and intervention periods depend on the step that has been randomized to the ward (Figure 1). Nevertheless, we will observe HCWs' hand hygiene compliance every month (every time-point).

Post randomization study visits will be divided in **ward visits** and **HCWs visits** (Figure 3)**.** In total we will perform a minimum of 16 visits per ward, 8 at the wards level and 8 at the individual HCW

level. Ward visits, performed every 4 weeks, are intended to monitor the study implementation and collect data at ward level; these visits will be performed preferentially by the investigator not in charge of the hand hygiene observations. HCWs visits, also performed every 4 weeks, are intended primarily to perform hh observations and will be performed by hand hygiene observers. Bellow a detailed description of visits

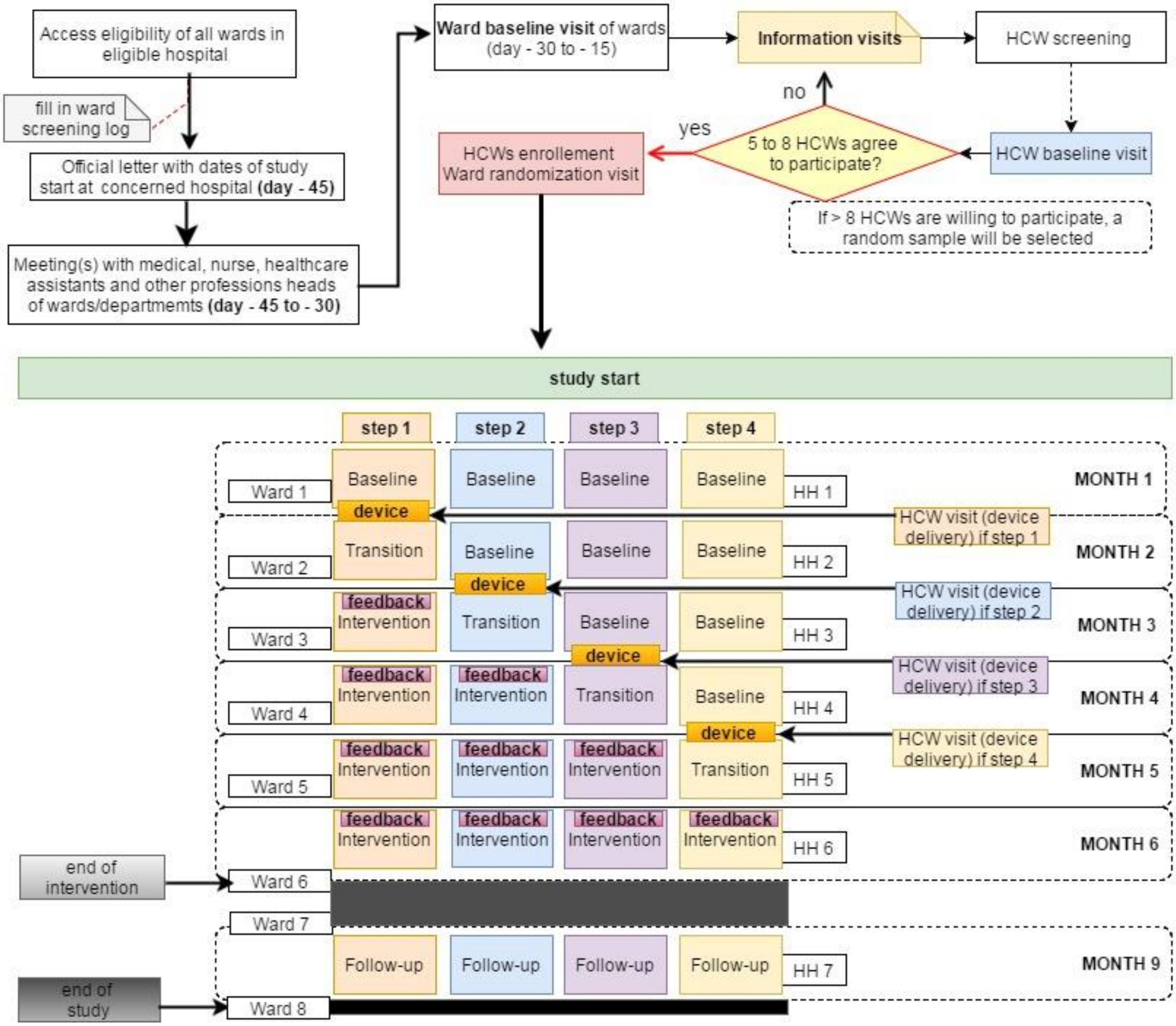

at ward and HCW level.

Figure 3: Schema of planned study visits. (device: when the device is delivered to the ward; feedback: when the device starts providing feedback to the HCW using it.)

Ward pre-randomization visits:

1. Ward baseline visit (- 4 weeks of study start): This visit will be made to the eligible wards. It will be held with the head nurse, and it can also be held with the head of physicians, head of dieticians and physiotherapists. The objectives of this visit are to explain the study, provide study information leaflets, confirm ward eligibility, access eligibility of HCWs working in the ward (**fill in HCW screening log**) and schedule information visits to the wards to recruit HCWs. Data will also be collected to CRF at the ward level (Table 2).

2. Information ward visits (- 4 weeks of study start to – 1 week of study start): These visits are intended to recruit HCWs for the study. It will be arranged in order to have the maximum HCWs present (possibly at transmission from the morning shift to the afternoon shift), of several professional categories. We intend this visit to last around 10 minutes. In this visit we will present the study (by showing the video prepared for this purpose, and by showing a small power point presentation), answer to questions and distribute study information leaflets. At the end of the presentation we will have **HCW screening visit** with HCWs that are interested to participate. If the HCW is eligible, a **HCW baseline visit** will be performed. The HCW screening log should be updated at the end of each information visit.

If with 2 information visits we are not able to recruit enough HCWs we will perform information visits until the date of study start in order to complete the recruitment. If 5 HCW are not recruited, we will include the unit with the available number of HCWs. After having at least 5 HCWs that have performed a baseline visit we will schedule a **ward randomization visit** with the head nurse.

3. Ward randomization visit (as from 5 days before the start of the study): During this visit HCWs inclusion criteria will be confirmed and it will be verified that all Informed Consents are signed. The randomization envelope will be opened at this time by the investigator not in charge of hand hygiene observations and its information placed safely (not in the CRF folder or the HCWs folder). The head nurse (HN), HCWs and HH observers will be blinded to the step the ward is allocated at least until one week before the start of the transition period in the said ward.

During this visit a ward and a HCW ID will be given.

The study investigators will define the future ward visit dates together with the HN head nurse and the HCWs planning (in order to organize the HH visits will be asked).

Study start /randomization (day 0)

Ward post-randomization visits:

4. Ward 1 (month 1 visit: week 4 after randomization): In all post-randomization ward visits (1 to 8), ward data will be collected, general issues related to the study implementation (as verifying that all hh observations are going as scheduled) will be addressed and working schedules of HCW will be obtained for the following month (in order to guide the timing of hh observation).
If the ward is allocated to step 1, it will be in this visit that the head nurse will be informed of the allocation of the ward in step 1 (this meaning that the following month his/her unit will pass to the transition period). In these ward visits (pre-transition period visits), the necessary arrangements will be made for the introduction of the device in the ward (as setting the location for the placement of the recharging station, etc). However, the devices and the charging station will only be brought to the ward in the first weekly day of the start of the transition period. The head nurse will be in charge of informing the HCWs that the device will be disponible in that day and will encourage HCWs to start using it. This visit is presential for step 1 but can be or not for step 2, 3 and 4 (study team can also contact by email or telephone the head nurse).

5. Ward 2 (month 2 visit: week 8 after randomization): If ward is allocated to step 2, it will be in this visit that the head nurse will be informed to the allocation of the ward in step 2 (this meaning that the following month his/her unit will pass to the transition period). This visit is presential for step 2 but can be or not for step 1, 3 and 4. If step 1 ward, inform that the ward will progress to the intervention period in the following month (this meaning that the feedback will be activated to HCWs).

6. Ward 3 (month 3 visit: week 12 after randomization): If ward is allocated to step 3, it will be in this visit that the head nurse will be informed to the allocation of the ward in step 3 (this meaning that the following month his/her unit will pass to the transition period). This visit is presential for step 3 but can be or not for step 1, 2 and 4. If step 2 ward, inform that the ward will progress to the intervention period in the following month (this meaning that the feedback will be activated to HCWs).

7. Ward 4 (month 4 visit: week 16 after randomization): If ward is allocated to step 4, it will be in this visit that the head nurse will be informed to the allocation of the ward in step 4 (this meaning that the following month his/her unit will pass to the transition period). This visit is presential for step 4 but can be or not for step 1, 2 and 3. If step 3 ward, inform that the ward will progress to the intervention period in the following month (this meaning that the feedback will be activated to HCWs).

8. Ward 5 (month 5 visit: week 20 after randomization): If step 4 ward, inform that the ward will progress to the intervention period in the following month (this meaning that the feedback will be activated to HCWs).

9. Ward 6 (month 6 visit: week 25 after randomization): End of intervention.

Ward follow-up period visits:

10. Ward 7 (+ 9 months after randomization): It should be verified that all HCWs are still working in this ward; if not state reasons for not working any more in the unit. This is the begin of follow-up.

11. Ward 8 (+ 10 months after randomization): End of study.

HCW pre-randomization visits:

1.HCWs screening visit: This visit will be the first HCW visit and intends to complete CRF HCW screening (basic demographics data), assess for study eligibility, give HCW study kit, give informed consent (long version) and schedule HCW baseline visit.

2. HCWs baseline visit: This visit is intended to confirm the intention of HCWs to participate in the study, to obtain the signed informed consent (mandatory) and to collect baseline data. Additionally, a detailed description of the study design will be made and a demonstration of how the device works will be performed.

Study start /randomization (day 0)

HCW post-randomization visits:

Investigators in charge of the HH observations will keep a daily updated file of HH observations. The HH auditors will organize the HH observations in order to obtain a homogenous sample of all the wards in all weeks of observation. An exception to this rule is if the HCW is on a ward on transition period or the first month of the intervention period. If this is the case, the HCW will only be observed regarding hand hygiene compliance from the second week of the respective period. This is intended to account for what the investigators considered to be a “implementation lag” of the device use (ie., so that the HCW will not be observed after using the device for one or 2 days).

If more than 2 tentatives to observe the HCW do not result in a HH observation session, the head nurse will be contacted and a specific schedule will be arranged.

In order to re-explain briefly the device use, an **extra HCW visit** will be performed with each HCW on their first working day after the device has been introduced to the ward (this corresponds to the begin of the transition period).

Of note, we don't intend to give personal feedback regarding hand hygiene compliance after each session of observation. The HH monitoring is only to collect data not perform feedback.

3. HCW HH1 (from week 1 to 4 after randomization): HH observation session (at least 5 HH opp for each HCW).

4. HCW HH2 (from week 6 to 8 after randomization): HH observation session (at least 5 HH opp for each HCW). If the HCW is in a ward allocated to step 1 (meaning that she/he will begin the transition period this month), a brief extra visit will take place on the first working day of this month to re-explain the use of the device.

5. HCW HH3 (from week 10 to 12 after randomization): HH observation session (at least 5 HH opp for each HCW). If the HCW is in a ward allocated to step 2 (meaning that she/he will begin the transition period this month), a brief extra visit will take place on the first working day of this month to re-explain the use of the device. If the HCW is allocated to step 1, the device will start providing feedback in the beginning of this month.

6. HCW HH4 (from week 14 to 16 after randomization): HH observation session (at least 5 HH opp for each HCW). If the HCW is in a ward allocated to step 3 (meaning that she/he will begin the transition period this month), a brief extra visit will take place on the first working day of this month to re-explain the use of the device. If the HCW is allocated to step 2, the device will start providing feedback in the beginning of this month.

7. HCW HH5 (from week 18 to 20 after randomization): HH observation session (at least 5 HH opp for each HCW). If the HCW is in a ward allocated to step 4 (meaning that she/he will begin the transition period this month), a brief extra visit will take place on the first working day of this month to re-explain the use of the device. If the HCW is allocated to step 3, the device will start providing feedback in the beginning of this month.

8. HCW HH6 (from week 22 to 24 after randomization): HH observation session (at least 5 HH opp for each HCW). If the HCW is allocated to step 4, the device will start providing feedback in the beginning of this month.

HCW follow-up period visits:

9. HCW HH7 (from 9 months to 10 months after randomization): HH observation session (at least 5 HH opp for each HCW).

13. Data collection

<u>Ward data collection</u>:

Ward screening visit (to be collected on **ward screening log**): date of visit, name of hospital, name of ward, screening number of ward, type of ward (ger, rehab, medical, surgical), assess and document exclusion criteria and/or reason for no participation (if given), if eligible: number of enrolment and date of baseline visit and contacts.

Table 2: Ward data to be completed in the ward CRFs and the ward dossier font.

Table 2: Ward data to be completed in the ward CRF and the ward dossier font. **Ward baseline**	Date of visit<br>ID of hospital<br>ID ward<br>General ward data:<br>- type of ward: surgical, medical, geriatrics, rehabilitation<br>- number of hospital beds at the study start<br>- bed occupancy in last calendar year<br>- ward HH compliance in last calendar year (if not available, collect at department level)<br>- ward ABHR consumption in last calendar year (ABHR litres dispensed by pharmacy per 1000 patient/days; if not available, collect at department level)<br><br>*Other (to be completed in the dossier source or other, not CRF):*<br>- Ward name, hospital name<br>- Confirm ward eligibility<br>- Complete name and contacts of head nurse, head physician, head of dieticians, head of physiotherapists (on screening page)<br>- Fill in HCWs overall screening log<br>- Schedule Information visits to wards<br>- Give ward study brochure / timeline
**Ward information**	No data collected to CRF<br><br>*Other (to be completed in the dossier source or other, not CRF):*<br>- Collect data on how many HCWs and professional category were present in information visits<br>- Fill in HCWs screening log<br>- Distribute HCWs study brochure and informed consent to HCW willing to participate<br>- Schedule HCWs baseline visit

**Ward randomization**	Date of visit<br>ID of hospital<br>ID of ward<br><br>- ID of ward<br>- ID of HCWs enrolled<br>- Date of device use start (it will be coded for data analysis)<br>- Date of feedback start (it will be coded for data analysis)<br>- Date of end of intervention (it will be coded for data analysis)<br>- Date of end of study (it will be coded for data analysis)<br><br>*Other (to be completed in the dossier source, not CRF):*<br>- Confirm absence of HCWs exclusion criteria and perform random sampling of 8 HCW if more are eligible to participate<br>- attribute ward ID and HCW ID<br>- Fill in ward ID doc and HCW ID doc<br>- Open randomization envelop (note on confidential sheet)<br>- Obtain HCWs schedule for study start month (if available)<br>- Schedule ward 1 to 6 visits<br>- Date of device use start<br>- Date of feedback start<br>- Date of end of intervention<br>- Date of end of study
**Ward 1 to 6** (to be recorded in each visit)	Date of visit<br>ID of hospital<br>ID of ward<br>- Type of visit (presencial or not – email telephone, none)<br>- ward hand hygiene compliance per month (number of hh opp, number of hh actions, number of HCWs observed, number of hh sessions)<br>- ward ABHR consumption per month (ABHR L dispensed by pharmacy per 1000 patient/days)<br>- number of days (considered as 8h shift) of each individual HCW work according to planning/corrected by head nurse (for adherence measure)<br>- number of patient-days per month (patient-days/month)<br><br>*Other (to be completed in the dossier source, not CRF):*<br>No data

**Ward 7 follow-up**	Date of visit<br>ID of hospital<br>ID of ward<br>- IDs of HCWs randomized still working in the ward<br><br>*Other (to be completed in the dossier source, not CRF):*<br>- Reasons for not being able to perform follow-up of HCWs
**Ward 8 end of study**	Date of visit<br>ID of hospital<br>ID of ward

<u>HCW data collection</u>:

HCW screening visit (to be collected on **HCW screening log**): name of hospital; name of ward; name of HCW; screening number of HCW; date of screening; month and year of birth, gender, profession; assess and document exclusion criteria and/or reason for no participation (if given); if absence of exclusion criteria and willing to participate (eligible): date of baseline visit.

Table 3: HCW data to be completed in the HCW CRFs and the HCW dossier font.

**HCW baseline**	Date of visit<br>ID of hospital<br>ID of ward<br>ID of HCW<br>General HCW data:<br>- (Month) and year when started working in healthcare (excluding the years of formation)<br>- (Month) and year when started of last hand hygiene +- IPC training (at least 1 hour structured training on IPC and hand hygiene) (excluding the years of formation) (at baseline visit dossier source, with HCW)<br>- Working schedule (full-time, part-time, if part time, what % ?)<br>- Hand size (in cm)<br>- Hand size (category: small, medium, large)<br>- ABHR volume personalized<br><br>*Other (to be completed in the dossier source, not CRF):*<br>Confirm eligibility<br>Informed consent signed<br>Hopirub rinse, hopirub gel, sterilium<br>Colour of device
**HCW inclusion**	Date of visit<br>ID of ward<br>ID of HCW<br>If not enrolled state why<br>Randomization number/ID of HCW

**HCW visits 1 to 7**	Date of visit<br><br>ID of ward<br><br>ID of HCW<br><br>Data extracted directly form tablet database (considered CRF):<br>- Date (dd/mm/yy) of hand hygiene observation<br>- Time (hh:mm), to be categorized in early morning (from 7h to 8h), morning (between 9 and 12h), early afternoon (from 13h to 14h), afternoon (from 15h to 19h), night (from 20h to 6h)<br>- Indications for HH (5 moments)<br>- Number of hh opportunities observed<br>- Number of hh actions performed<br>- type of hh action (ABHR vs hand washing)<br>- feedback provided to HCWs (yes/no)<br><br>To be collected on paper CRF:<br>- adverse events (open response, to be asked each time a HCW with the device is observed after a HH session and registered at the dossier source HH) – skin irritation, injury to patient, etc).<br><br>*Other (to be completed in the dossier source, not CRF):*<br>Comments
**HCW device delivery visit**	Date of visit<br><br>ID of ward<br><br>ID of HCW<br><br>Date of first device utilization *(also registered by device automatically)*<br>Date of planned device feedback start / state if confirmed *(also registered by device automatically)*<br>Identification of attributed device bottle<br>Identification of attributed device bracelet<br>*Other (to be completed in the dossier source, not CRF):*<br>Comments

**Data collected by device continuously**	ID of ward<br>ID of HCW<br>Data collected by the device (device database considered CRF):<br>- Code of bottle and bracelet of device<br>- dd/mm/yy and of hh:mm:ss ABHR use<br>- dd/mm/yy and hh:mm:ss of hand friction<br>- volume of ABHR used (ml)<br>- duration of hand friction (sec)<br>- feedback provided on volume (yes/no)<br>- feedback provided on duration (yes/no)<br>- number of device-days of use (considered as approximately 8h shift) (device-days)<br>- number of device-hours of use (device-hours)
**HCW visit 8**	ID of ward<br>ID of HCW<br>Questionnaire<br>*Other (to be completed in the dossier source, not CRF):*<br>Comments

14. Primary and secondary endpoints

The **primary outcome** is the performance of HH action by the HCW observed in the presence of an opportunity for hand hygiene during the course of patient care (HH compliance). Hand hygiene compliance will be measured at the individual HCW (closed cohort) level at 6 time-points (once a month) in the 3 study periods (baseline, transition period and intervention) and at follow-up. For this outcome, we will consider a HH opportunity as a binary variable, to be taken as a HH action or not, by the HCW. The primary outcome will be defined by the observer who will also define the opportunity (according to standard procedures at HUG)[6,11,14] through “on site” direct observation by well-trained IPC professionals from SPCI directly on a electronic device (tablet). The method of HH observation was developed by the HUG team 20 years ago and has been routinely used since then, both for clinical and research purposes, with excellent inter-observer agreement (kappa > 80%).[6,11,14] The method consists of an observation of a HCW during a 20 minutes session and the anonymous record of each HH opportunity and its correspondent action or the absence of action. Each HH opportunity is classified according to the WHO’s 5 moments, as previously described.[12] Each month the investigators in charge of HH observations will collect at least 5 and 10 opportunities per HCW. Observations will be performed during day working hours and during weekdays (night shifts and weekends are excluded). A schedule of visits which takes

into account the HCWs working schedule will be compiled and observations will be performed in all participating wards throughout the month (to ensure a homogenous sample for all wards throughout each week of the month). Additionally, HCWs carrying the device with/ or without feedback for the first time will not be observed during the first week of the respective month. This is intended to account for a "implementation lag" of the device.

The **secondary outcomes** were divided in 8 levels:

- **Hand hygiene quality**: volume of ABHR poured by HCWs and the duration of handrubbing in each HH action, using the device without feedback (transition period) or device with feedback (intervention period). This data will be collected automatically and continuously by the device (CRF device database). Finally, the quality of HH action, assessed by the use of the two previous secondary outcomes, will be evaluated. HH action will be defined as "of quality" if HH action includes the use of the minimum personally specified amount of ABHR and if it lasts at least 15 seconds; otherwise it will be defined as "not adequate". This outcomes will not be evaluated at baseline and follow-up since HCWs will not use the device during these periods.
- **Frequency of hand hygiene action**: data on the frequency of HH action as recorded by the device on the transition and intervention period. This data will be collected automatically and continuously by the device (CRF device database). Sub-analysis will be performed according to the period of the day, type of ward, presence of hand hygiene observers, etc.
- **Hand hygiene compliance at follow-up**: hand hygiene compliance will be measured 3 months after the intervention stops to assess for the sustainability of the intervention;
- **Hand hygiene compliance and alcohol-base handrub consumption at the ward level**: HH compliance data is recorded at a regular basis by the IPC professionals and ABHR consumption is provided monthly by the pharmacy (monitored by dividing monthly ward-specific requisition of alcohol-based handrub by 1000 patient-days; only 100 mL bottles of alcohol-based handrub carried by health-care workers for their personal use are included in the measure, because this is the predominant means of hand hygiene among HCWs).
- **Adherence to use of hand hygiene device**: we will measure how many hours the device will be used by HCWs (provided directly by the device, recorded as time of device out of the station), adjusted then for days of use (as working shifts are 8 h, we will consider a 6h use as a day use). Days of device use will be divided by HCWs working days, and obtain a proportion of device days use by HCW.
- **Satisfaction and perception of usefulness of the device by HCWs**: a questionnaire and focus groups discussion will be performed with HCWs participating in the study to evaluate their experience with the utilization. This will be performed at the end of the intervention.

- **Hand hygiene quality and HH compliance among HCWs working in several units** during the study period. This is done as a sub-study with HCWs that are willing to participate but that are excluded from the main study due to working in several units (meaning that they can't be allocated to a cluster). This group of HCWs receives the device only in the fifth month of the study, when all the units have already started the study intervention, to avoid contamination. These HCWs data does not contribute to the primary outcome analysis.
- **Adverse events** related to the device are measured using a list of open responses (ie., skin irritation, injury to patient, etc) asked to participants after each HH session
- **Bloodstream infections (BSI)** surveillance are measured using the data routinely and prospectively collected by the IPC program in order to analyse if there is a change in incidence of BSI in the units during the study period.

15. Study population & Eligibility criteria

Study population: healthcare workers working at the University Hospitals of Geneva (HUG) with patient care activities.

- All **units** will be screened to participate in the study.

Inclusion criteria: All units are eligible to participate in the study.
Exclusion criteria: Units without patient-care activities.

- All **HCWs** in eligible wards can participate in the study.

Inclusion criteria: All HCWs working in patient-care activities.
Exclusion criteria:
1. Work or will work in several different units during the six months after study start
2. Who will leave the unit in the six months after study start
3. Who have more than three consecutive weeks of vacations in the six months after the study start
4. Who don't use the standard ABHR at HUG

16. Intervention: the electronic device

The intervention consists of wearing an electronic device in the form of a wrist band and a bottle of ABHR with a "clip" inside that provides a personal, automatic and individualized feedback on hand hygiene quality surrogate markers to HCWs. This device was developed at HUG in cooperation with the

School of Engineers of Geneva. Both components of the device are equipped with electronic sensors that continuously record data on the volume of ABHR used (clip in the bottle) and the duration of hand friction (wrist band). These components communicate with each other and transmit the information to a computer, cell phone or tablet equipped with the designated software.

When the feedback option of the device is activated, the device can provide automatic feedback to the user regarding the quality of HH action, based on the volume of ABHR used and the duration of each HH action. This is a positive feedback and consists in a vibration: the personal bottle of ABHR cam vibrate when a previously determined volume of ABHR[79] is poured and the bracelet can vibrate each time hand friction (handrubbing) lasts for a pre-determined amount of time (15 seconds).[86]

The volume of ABHR at which the device (bottle) will be set to provide feedback will be determined individually for each participant, based on their own hand surface area.[85] This will be calculated according to the formula that has been derived from the laboratory studies on volume of ABHR[85] and aiming for a 2 log bacterial reduction in HCWs hands (model without interaction between the category of hand size and the volume; Table 4)

Table 4: Calculation of the personalized volume of ABHR to be used in the device (according to hand size category)

Hand size category	Small hands	Medium hands	Large hands
Mean volume of ABHR for a 2 log reduction (mL)	2.2 mL	2.3 mL	3.3 mL
Mean hand surface (cm^2)	332.93cm^2	404.20cm^2	473.2 cm^2
Volume of ABHR per cm^2 (mL/ cm^2)	0.0066 mL/cm^2	0.0057 mL/cm^2	0.007 mL/cm^2

However, if according to this calculation, the volume of ABHR is less than the mean volume that is normally used by the HCW (and measured during the transition period) we will use the mean volume normally used by the HCW to set the feedback of the device.

The duration of hand friction at which the device (bracelet) will be set to give a feedback is 15 seconds. This was also based on a recent publication form our team.[86] Again, if the mean duration of hand friction measured during the transition period is higher than 15 seconds, we will use the mean duration of hand friction already done by the HCW to set the feedback of the device.

Apart from providing this automatic feedback, the device also records the number of HH actions by HCW, the date and time of performing the HH action, the volume of ABHR per HH action and the duration of hand friction. Data collection will also include the total amount of time of device use by the HCW.

Data from the device is recovered by electronic sensors from a portable station that will be placed in each participating ward. In this station HCWs will position their devices when these are not in use, Consecutively, the station will transfer the information to a server computer, equipped with an appropriate homemade software. This data will be available for study investigators at real time on a website. The ID of HCWs will be coded thus guarantying that the data is anonymous.

During the baseline visit HCWs will be shown the device and instructed on how to use it. On the beginning of the transition phase (where HCWs will be provided with the device), the investigators of the study (not involved in HH observations) will conduct another HCW visit reminding HCWs on how to use the device. A telephone line will be continuously made available to assist users for any questions or difficulties. A 'good practice' leaflet on the device use and a poster with clear instructions on use and decontamination procedure will be made available near each device station. The device meets the necessary requirements for use in a healthcare setting since it is smooth, ergonomic, cleanable, and can be easily disinfected with disinfectants like alcohol or polihexametilbiguanida.

17. Safety

Data will be collected regarding adverse events related to skin sensibility, patient harm, etc. It will be recorded as a secondary outcome during each ward visit when observing individual HCWs.

18. Withdrawal process

HCWs can withdrawal without notice when they want.

19. Data management

Source data: source data are screening log (hospital, ward and HCW), the dossier font (ward and HCW); hospital ID, ward ID and HCW ID codes. Allocation of steps to wards list.

CRF: paper CRF (ward and HCW), electronic CRF of hand hygiene observations and electronic CRF of device data collection.

Document storage and keeping: Data collected in CRFs will be introduced into a secured excel database. Data directly collected on the tablet (hand hygiene compliance) and by the electronic device will be stored in secured servers.

20. Monitoring

There will be no external monitoring in this study.

21. Criteria for ending of trial / definition of end of trial

The trial will end after the follow-up period (end of February 2018).

22. Ethical considerations

The study protocol was submitted to the Regional Research Ethics Committee (CCER), Geneva, Switzerland, 08/06/2016, ref: (2016-00714). Informed written consent will also be required from each HCW participating in the study. The study protocol is registered at the ISRCTN25430066, 22/05/2017 (http://www.isrctn.com/ISRCTN25430066).

Although the use of ABHR may cause minor skin reactions, the same product is currently being used in all clinical settings at HUG, so the present study poses no additional risk to HCWs. All data collected will be anonymous and none of the study procedures will interfere with patient care. In addition, the device complies with current legislation requirements for a medical device used in the hospital setting. Heads of the concerned departments and wards will be informed of the objectives and interventions planned in the context of this study.

23. Timeframe

The protocol will be implemented from May 2017 until February 2018. The data entry will be performed at the same time. We will perform the data cleaning and analyze the data from February 2018 to April 2018. After that we will present the results on national and international conferences, as well as write a manuscript for submission.

24. Limitations

The vast majority of HCWs directly involved in patient care are assigned to work in a single ward. However, others – mainly physicians, physiotherapists etc.. – perform their activities amongst various wards. This situation may lead to some form of 'contamination', which might decrease the inter-cluster

variation and thus affect the study power (however, this is an exclusion criteria so we hope this situation will be residual). Moreover, the HH opportunities performed by physicians are much lower than HH accomplished by nurses. Thus, contamination of the clusters due to migration of HCWs is negligible. Statistical power: planning of cluster-randomised trials requires the estimation of an a priori ICC value in order to evaluate sample size. The magnitude of this coefficient has a major impact on the power of the study. Thus, our estimate of ICC has been conservative so to reduce the risk of type II error. However, previous studies have shown that a priori ICC values are not completely reliable. Thus, the risk of type II error may be significant despite conservative estimations. The CONSORT statement for cluster-randomised trials[92] recommends the estimation of an intermediate ICC value during the trial to allow for a sample size adjustment. This study will comply with the CONSORT recommendation.

25. Importance and impact

Due to the exponential increase of antimicrobial resistance amongst bacterial pathogens, their transmission in hospitals predominantly through HCWs hands, and due to the global burden of HAI worldwide, HH is more important now than ever. In this regard, the quality of HH action is considered to be as relevant as compliance. The device intended to be studied in the current proposal may improve both compliance and quality of HH action in a continuous manner, and ultimately contribute to reduce HAI and antimicrobial resistance spread in all type of health care settings, eventually helping to make hospitals a safer place to be, as a patient or a HCW.

26. References

1. Allegranzi B, et al. Burden of endemic health-care-associated infection in developing countries: systematic review and meta-analysis. Lancet. 2011 Jan 15;377(9761):228-41.
2. European Centre for Disease Prevention and Control. Point prevalence survey of healthcare associated infections and antimicrobial use in European acute care hospitals. Stockholm: ECDC; 2013. Available at: http://www.ecdc.europa.eu/en/publications/Publications/healthcare-associated-infections-antimicrobial-use-PPS.pdf
3. European Centre for Disease Prevention and Control: Annual Epidemiological Report on Communicable Diseases in Europe 2008. Stockholm, European Centre for Disease Prevention and Control, 2008. Available at: http://www.ecdc.europa.eu/en/publications/Publications/0812_SUR_Annual_Epidemiological_Report_2008.pdf
4. Office Fédéral de la Santé Publique. [Stratégie nationale de surveillance, de prévention et de lutte contre les infections HAIes (stratégie NOSO): Foire aux questions], 2014.
5. Pittet D, et al. Evidence-based model for hand transmission during patient care and the role of improved practices. Lancet Infect Dis 2006; 6: 641–652.
6. Pittet D, et al. Effectiveness of a hospital-wide programme to improve compliance with hand hygiene. Lancet, 2000. 356: 1307-1312.
7. Pittet D, Compliance with hand disinfection and its impact on hospital-acquired infections. J Hosp Infect, 2001. 48 (Suppl A): S40-6.
8. Ling ML, How KB. Impact of a hospital-wide hand hygiene promotion strategy on healthcare-associated infections. Antimicrob Resist Infect Control 2012;1:13.
9. Loveday HP, et al. epic3: national evidence-based guidelines for preventing healthcare-associated infections in NHS hospitals in England. J Hosp Infect 2014;86(Suppl 1):S1-70.
10. Ellingson K, et al. Strategies to prevent healthcare-associated infections through hand hygiene. Infect Control Hosp Epidemiol 2014;35:937-60.
11. Hugonnet S, et al. Alcohol-based handrub improves compliance with hand hygiene in intensive care units. Arch Intern Med. 2002 May 13;162(9):1037-43.
12. WHO. WHO Guidelines on Hand Hygiene in Health Care, 2009, World Health Organization: Geneva. Available at: http://apps.who.int/iris/bitstream/10665/44102/1/9789241597906_eng.pdf

13. WHO. Guide to Implementation: A Guide to the Implementation of the WHO Multimodal Hand Hygiene Improvement Strategy, 2009. Available at: http://whqlibdoc.who.int/hq/2009/WHO_IER_PSP_2009.02_eng.pdf
14. Sax H et al. The World Health Organization hand hygiene observation method. Am J Infect Control 2009;37:827-834.
15. Zahar JR. Is hand-rub consumption correlated with hand hygiene and rate of extended-spectrum beta-lactamase-producing Enterobacteriaceae (ESBL-PE)-acquired infections? J Hosp Infect. 2012 Apr;80(4):348-50.
16. Vernaz N et al. Temporal effects of antibiotic use and hand rub consumption on the incidence of MRSA and Clostridium difficile. J. Antimicrob. Chemother. (2008) 62 (3): 601-607.
17. Sax H et al. 'My five moments for hand hygiene': a user-centred design approach to understand, train, monitor and report hand hygiene. J Hosp Infect. 2007 Sep;67(1):9-21.
18. Allegranzi B, et al. Status of the implementation of the World Health Organization multimodal hand hygiene strategy in United States of America health care facilities. Am J Infect Control. 2014 Mar;42(3):224-30.
19. Salmon S, et al. Beginning the journey of hand hygiene compliance monitoring at a 2,100-bed tertiary hospital in Vietnam. Am J Infect Control. 2014 Jan;42(1):71-73.
20. Allegranzi B, et al. Global implementation of WHO's multimodal strategy for improvement of hand hygiene: a quasi-experimental study. Lancet Infect Dis. 2013 Oct;13(10):843-851.
21. Barrera L, et al. Effectiveness of a hand hygiene promotion strategy using alcohol-based handrub in 6 intensive care units in Colombia. Am J Infect Control. 2011 Oct;39(8):633-9.
22. Lee A, et al. Hand hygiene practices and adherence determinants in surgical wards across Europe and Israel: a multicenter observational study. Am J Infect Control. 2011 Aug;39(6):517-520.
23. Mathai E, et al. Promoting hand hygiene in healthcare through national/subnational campaigns. J Hosp Infect. 2011 Apr;77(4):294-298.
24. Stewardson A, et al. Quicker, easier, and cheaper? The promise of automated hand hygiene monitoring. Infect Control Hosp Epidemiol. 2011 Oct;32(10):1029-1031.
25. Stewardson A, et al. Impact of observation and analysis methodology when reporting hand hygiene data. J Hosp Infect. 2011 Apr;77(4):358-359.
26. Dhar S, et al. Observer bias in hand hygiene compliance reporting. Infect Control Hosp Epidemiol 2010;31(8):869-70.
27. D'Egidio G, et al. A study of the efficacy of flashing lights to increase the salience of alcohol-gel dispensers for improving hand hygiene compliance. Am J Infect Control 2014;42(8):852-5.

28. Fakhry M, et al. Effectiveness of an audible reminder on hand hygiene adherence. Am J Infect Control 2012;40(4):320-3.
29. Fisher DA, et al. Automated measures of hand hygiene compliance among HCWs using ultrasound: validation and a randomized controlled trial. Infect Control Hosp Epidemiol 2013;34(9):919-28.
30. Marra AR, Edmond MB. New technologies to monitor HCW hand hygiene. Clin Microbiol Infect 2014;20(1):29-33.
31. Scheithauer S, et al. Influence of signal colored hand disinfectant dispensers on hand hygiene compliance at a medical intensive care unit. Am J Infect Control 2014;42(8):926-8.
32. Levchenko AI, et al. Automated monitoring: a potential solution for achieving sustainable improvement in hand hygiene practices. Comput Inform Nurs 2014;32(8):397-403.
33. Muller MP, et al. Electronic monitoring of individual HCWs' hand hygiene event rate. Infect Control Hosp Epidemiol 2014;35(9):1189-92.
34. Storey SJ, et al. Effect of a contact monitoring system with immediate visual feedback on hand hygiene compliance. J Hosp Infect 2014.
35. Boudjema S, et al. MediHandTrace (R): a tool for measuring and understanding hand hygiene adherence. Clin Microbiol Infect 2014;20(1):22-8.
36. Marra AR, et al. The use of real-time feedback via wireless technology to improve hand hygiene compliance. Am J Infect Control 2014;42(6):608-11.
37. Ward MA, et al. Automated and electronically assisted hand hygiene monitoring systems: a systematic review. Am J Infect Control 2014;42(5):472-8.
38. Davis CR. Infection-free surgery: how to improve hand-hygiene compliance and eradicate methicillin-resistant Staphylococcus aureus from surgical wards. Ann R Coll Surg Engl 2010;92:316-9.
39. Armellino D, et al. Using high-technology to enforce low-technology safety measures: the use of third-party remote video auditing and real-time feedback in healthcare. Clin Infect Dis 2012;54:1-7.
40. Armellino D, et al. Replicating changes in hand hygiene in a surgical intensive care unit with remote video auditing and feedback. Am J Infect Control 2013;41:925-927.
41. Palmore TN, Henderson DK. Big brother is washing. Video surveillance for hand hygiene adherence, through the lenses of efficacy and privacy. Clin Infect Dis 2012;54:8-9.
42. Boyce JM. Electronic Monitoring in Combination with Direct Observation as a Means to Improve Hand Hygiene Compliance. *American Journal of Infection Control* (in press).
43. Pires D., Pittet D. Hand hygiene electronic monitoring: are we there yet? *American Journal of Infection Control* (in press).

44. Larson EL, et al. An organizational climate intervention associated with increased handwashing and decreased nosocomial infections. Behav Med 2000;26:14-22.
45. Larson EL, et al. Hand hygiene behavior in a pediatric emergency department and a pediatric intensive care unit: comparison of use of 2 dispenser systems. Am J Crit Care 2005;14:304-11.
46. Kinsella G, et al. Electronic surveillance of wall-mounted soap and alcohol gel dispensers in an intensive care unit. J Hosp Infect 2007; 66:34-9.
47. Whitby M, et al. Three successful interventions in health care workers that improve compliance with hand hygiene: is sustained replication possible? Am J Infect Control 2008;36:349-55.
48. Boyce JM, et al. Evaluation of an electronic device for real-time measurement of alcohol-based hand rub use. Infect Control Hosp Epidemiol 2009;30:1090-5.
49. Koff MD, et al. Reduction in ventilator associated pneumonia in a mixed intensive care unit after initiation of a novel hand hygiene program. J Crit Care 2011;26:489-95.
50. Koff MD, et al. Reduction in intraoperative bacterial contamination of peripheral intravenous tubing through the use of a novel device. Anesthesiology 2009;110:978-85.
51. Marra AR, et al. Controlled trial measuring the effect of a feedback intervention on hand hygiene compliance in a step-down unit. Infect Control Hosp Epidemiol 2008;29:730-5.
52. Marra AR, et al. Positive deviance: a new strategy for improving hand hygiene compliance. Infect Control Hosp Epidemiol 2010;31:12-20.
53. Marra AR, et al. Measuring rates of hand hygiene adherence in the intensive care setting: a comparative study of direct observation, product usage, and electronic counting devices. Infect Control Hosp Epidemiol 2010;31:796-801.
54. Marra AR, et al. Positive deviance: a program for sustained improvement in hand hygiene compliance. Am J Infect Control 2011;39:1-5.
55. Macedo RC, et al. Positive deviance: using a nurse call system to evaluate hand hygiene practices. Am J Infect Control 2012;40:946-50.
56. Helder OK, et al. Computer screen saver hand hygiene information curbs a negative trend in hand hygiene behavior. Am J Infect Control 2012;40:951-4.
57. Helder OK, et al. Hand disinfection in a neonatal intensive care unit: continuous electronic monitoring over a oneyear period. BMC Infect Dis 2012;12:248.
58. Morgan DJ, et al. Automated hand hygiene count devices may better measure compliance than human observation. Am J Infect Control 2012;40:955-9.
59. Boscart VM, et al. Advanced technologies to curb healthcare-associated infections. Healthc Pap 2009;9:51-5.

60. Swoboda SM, et al. Electronic monitoring and voice prompts improve hand hygiene and decrease nosocomial infections in an intermediate care unit. Crit Care Med 2004;32:358-63.
61. Swoboda SM, et al. Isolation status and voice prompts improve hand hygiene. Am J Infect Control 2007;35:470-6.
62. Venkatesh AK, et al. Use of electronic alerts to enhance hand hygiene compliance and decrease transmission of vancomycin-resistant Enterococcus in a hematology unit. Am J Infect Control 2008;36:199-205.
63. Sahud AG, et al. Measuring hand hygiene compliance: a new frontier for improving hand hygiene. Infect Control Hosp Epidemiol 2009;30:1132.
64. Sahud AG, et al. An electronic hand hygiene surveillance device: a pilot study exploring surrogate markers for hand hygiene compliance. Infect Control Hosp Epidemiol 2010;31:634-9.
65. Sahud AG, et al. Feasibility and effectiveness of an electronic hand hygiene feedback device targeted to improve rates of hand hygiene. J Hosp Infect 2012;82:271-3.
66. Boscart VM, et al. Acceptability of a wearable hand hygiene device with monitoring capabilities. J Hosp Infect 2008;70:216-22.
67. Levchenko AI, et al. The feasibility of an automated monitoring system to improve nurses’ hand hygiene. Int J Med Inform 2011; 80:596-603.
68. Cheng VC, et al. Introduction of an electronic monitoring system for monitoring compliance with Moments 1 and 4 of the WHO “My 5 Moments for Hand Hygiene” methodology. BMC Infect Dis 2011;11:151.
69. Polgreen PM, et al. Method for automated monitoring of hand hygiene adherence without radio-frequency identification. Infect Control Hosp Epidemiol 2010;31:1294-7.
70. Fries J, et al. Monitoring hand hygiene via human observers: how should we be sampling? Infect Control Hosp Epidemiol 2012;33:689-95.
71. Hornbeck T, et al. Using sensor networks to study the effect of peripatetic HCWs on the spread of hospital-associated infections. J Infect Dis 2012;206:1549-57.
72. Sharma D, et al. The precision of human-generated hand-hygiene observations: a comparison of human observation with an automated monitoring system. Infect Control Hosp Epidemiol 2012;33:1259-61.
73. Edmond MB, et al. Successful use of alcohol sensor technology to monitor and report hand hygiene compliance. J Hosp Infect 2010;76:364-5.
74. Joch A. Badge brings automated edge to infection control. Interview by Alan Joch. Mater Manag Health Care 2009;18:15-17.

75. Szilágyi1 L, et al. A large-scale assessment of hand hygiene quality and the effectiveness of the "WHO 6-steps". BMC Infectious Diseases 2013, 13:249.
76. Stewardson A, et al. Efficacy of a New Educational Tool to Improve Handrubbing Technique amongst HCWs: A Controlled, Before-After Study. PLoS ONE 2014 9(9): e105866.
77. Higgins A, et al. Improved hand hygiene technique and compliance in HCWs using gaming technology. Journal of Hospital Infection, 84 (2013) 32-37.
78. Laustsen S, Lund E, Bibby BM, Kristensen B, Thulstrup AM, Mller JK. Effect of correctly using alcohol-based hand rub in a clinical setting. *Infect Control* 2008;**29**(10):954–6.
79. Pittet D. Hand hygiene: it's all about when and how. *Infect Control Hosp Epidemiol* 2008;**29**(10):957–9. Doi: 10.1086/592218.
80. Boyce JM, Pittet D. Guideline for hand hygiene in health-care settings: recommendations of the Healthcare Infection Control Practices Advisory Committee and the HICPAC/SHEA/APIC/IDSA Hand Hygiene Task Force. *Am J Infect Control* 2002;**30**(8):S1–46.
81. European Committee for Standardization. European Norm 1500: chemical disinfectants and antiseptics. Hygienic handrub. Test method and requirements (phase 2/step 2). Version 2013.
82. Macinga DR, Shumaker DJ, Werner H-P, et al. The relative influences of product volume, delivery format and alcohol concentration on dry-time and efficacy of alcohol-based hand rubs. *BMC Infect Dis* 2014;**14**(1):1.
83. Gayet-Ageron A, Bellissimo-Rodrigues F, Soule H, Martin Y, Pittet D. Relationship between hand size, volume of alcohol-based handrub and time needed to dry hands. An experimental laboratory-based study. *Antimicrob Resist Infect Control* 2015;**4**(Suppl 1):P303. Doi: 10.1186/2047-2994-4-S1-P303.
84. Kampf G, Marschall S, Eggerstedt S, Ostermeyer C. Efficacy of ethanol-based hand foams using clinically relevant amounts: a cross-over controlled study among healthy volunteers. *BMC Infect Dis* 2010;**10**(1):1.
85. Bellissimo-Rodrigues F, et al. Should alcohol-based hand rub use be customized according to HCWs hand size? Submitted to the International Conference on Prevention and Infection Control (ICPIC 2015) on Mars 20, 2015. ICPIC15-ABS-1257.
86. Pires D., Soule H., Bellissimo-Rodrigues F., Gayet-Ageron A., Pittet D. Hand hygiene with alcohol-based handrub: how long is long enough? *Infect Control Hosp Epidemiol* (in press)
87. Dharan S., Hugonnet S., Sax H., Pittet D. Comparison of Waterless Hand Antisepsis Agents at Short Application Times: Raising the Flag of Concern •. *Infect Control Hosp Epidemiol* 2003;**24**(3):160–4. Doi: 10.1086/502182.

88. Sickbert-Bennett EE., Weber DJ., Gergen-Teague MF., Sobsey MD., Samsa GP., Rutala WA. Comparative efficacy of hand hygiene agents in the reduction of bacteria and viruses. *Am J Infect Control* 2005;**33**(2):67–77. Doi: 10.1016/j.ajic.2004.08.005.
89. Vesin JM et al. Recognition of hand gestures through accelerometer signals. Application to patient safety (2006-2009), Swiss national fund for scientific research.
90. Pittet D, et al. Hand hygiene among physicians: performance, beliefs, and perceptions. Ann Intern Med, 2004. 141(1): 1-8.
91. Hemming K, Haines TP, Chilton PJ, Girling AJ, Lilford RJ. The stepped wedge cluster randomised trial: rationale, design, analysis, and reporting. BMJ 2015;351:h39 doi: 10 .113 6/bmj.h391162.
92. Campbell MK, et al. Consort 2010 statement: extension to cluster randomised trials. BMJ 2012;345:e5661.
93. Allegranzi B, et al. Successful implementation of the World Health Organization hand hygiene improvement strategy in a referral hospital in Mali, Africa. Infect Control Hosp Epidemiol. 2010 Feb;31(2):133-41.
94. WHO. Hand Hygiene Technical Reference Manual. 2009. Available at: http://whqlibdoc.who.int/publications/2009/9789241598606_eng.pdf.
95. Woertman W, de HE, Moerbeek M, Zuidema SU, Gerritsen DL, Teerenstra S. Stepped edge designs could reduce the required sample size in cluster randomized trials. J Clin Epidemiol 2013;66:752e8.
96. Hemming K, Girling A. A menu driven facility for sample size for power and detectable difference calculations in stepped wedge randomised trials. Stata J 2014;14:363e80.
97. Baio G, Copas A, Ambler G, Hargreaves J, Beard E, Omar RZ. Sample size calculation for a stepped wedge trial. Trials 2015;16:354-368
98. Hemming K, Taljaard M. Sample size calculations for stepped wedge and cluster randomised trials: a unified approach. J Clin Epidemiol 2016;69:137-146.

99. White H. A heteroskedasticity-consistent covariance matrix estimator and a direct test for heteroskedasticity. Econometrica 1980; 48: 817–38; Williams RL. A note on robust variance estimation for cluster-correlated data. Biometrics 2000; 56: 645–46.).
100. European Medicines Agency (2015). Guideline on adjustment for baseline covariates in clinical trials. www.ema.europa.eu/docs/en_GB/document_library/Scientific_guideline/2015/03/WC500184923.pdf Accessed on 7 Mar 2017.

27. Appendice 1

Corrections applied to the clip to increase its precision and monitoring of the volume of ABHR used in a pilot study.

1) Corrections applied to the device:
Precision of the measures obtained in real time during patient care to hand disinfectant bottle :
The error distribution can be approximated by the following distribution (Figure 1) with parameters :

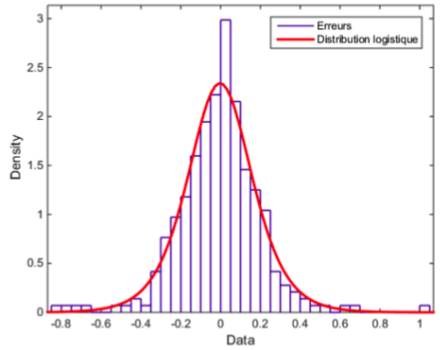

Note : 98% of the measures are in the error range [-0.5; 0.5] ml and 88% are in the range [-0.3; 0.3] ml.

2) Results from a pilot study monitoring the volume of ABHR used among HCW during patient care with vs. without device-provided feedback on volume use.
11 participants were included in the pilot to test in real life, during patient care, the effect of feedback to the individual HCW the volume of ABHR used. There were 4 periods without feedback and 4 periods with feedback provided. All 11 participants were included at the same time.
In the period without feedback (before feedback was given to the HCW) :

- 9 participants provided some data regarding the volume of ABHR used at time 1 (T1)
- 10 participants provided some data regarding the volume of ABHR used at T2
- 10 participants provided some data regarding the volume of ABHR used at T3
- 10 participants provided some data regarding the volume of ABHR used at T4

In the period with feedback provided:

- 9 participants provided some data regarding the volume of ABHR used at T1
- 9 participants provided some data regarding the volume of ABHR used at T2
- 7 participants provided some data regarding the volume of ABHR used at T3
- 8 participants provided some data regarding the volume of ABHR used at T4

The results are presented in the following Table.
In brief, the volume of ABHR used increased from a mean (±SD, median; p25-p75) of 1.33 ml (±0.37, 1.33; 1.07-1.54) without feedback to a mean of 3.63 ml (±0.87, 3.60; 3.04-4.11) after feedback.

Description of the duration, volume and number of disinfections used by the participants at each time-period

Description of the mean (±SD, median; p25-p75)	T1	T2	T3
Before (wo feedback)			
Time of observation (min.)	176.7 (±10; 180: 180-180)	324.0 (±110.3; 360: 300-420)	348.0 (±37.9; 360: 300-360)
Number of disinfection	14.3 (±7.5; 13: 8-18)	16.8 (±8.9; 18.5: 11-23)	20.1 (±9.0; 20.5: 12-27)
Volume of ABHR used	20.2 (±10.7; 20.9: 12.3-23.1)	22.2 (±13.5; 25.9: 7.5-32.3)	27.6 (±15.1; 28.7: 13-38.5)
Volume per action	1.40 (±0.40; 1.36: 1.28-1.61)	1.29 (±0.42; 1.26: 1.06-1.57)	1.33 (±0.39; 1.32; 1.08-1.54)
After (with feedback)			
Time of observation (min.)	286.7 (±40.0; 300: 300-300)	420 (±0; 420: 420-420)	360 (±0; 360: 360-360)
Number of disinfection	13.7 (±6.0; 15: 10-16)	18.6 (±5.1; 20: 19-22)	16.6 (±6.2; 18: 14-22)
Volume of ABHR used	53.8 (±21.6; 55.4: 43.9-62.6)	68.9 (±29.2; 63.4: 60-95.4)	50.0 (±19.1; 54.6: 26-62.3)
Volume per action	4.06 (±0.56; 3.96: 3.86-4.39)	3.73 (±1.21; 3.42: 3.34-4.63)	3.25 (±1.18; 3.19; 2.48-4.33)
P-values comparing after to before			
Time of observation	0.0174	0.0174	0.157
Number of disinfections	0.99	0.953	0.0277
Volume of ABHR used	0.0280	0.0077	0.0277
Volume per action	0.018	0.0077	0.0277

Description of the mean (±SD, median; p25-p75)	T4	Overall
Before (wo feedback)		
Time of observation (min.)	294.0 (±86.9; 240: 240-420)	1022.7 (±277.1; 1080: 780-1140)
Number of disinfection	9.2 (±6.6; 8.5: 5-10)	53.6 (±17.2; 56: 40-65)
Volume of ABHR used	12.9 (±9.4; 13: 3.4-15.6)	73.5 (±35.2; 60.3: 51.8-98.8)
Volume per action	1.32 (±0.38; 1.36: 1.13-1.52)	**1.33 (±0.37; 1.33: 1.07-1.54)**
After (with feedback)		
Time of observation (sec.)	90.0 (±55.5; 60: 60-120)	872.7 (±284.4; 780: 660-1140)
Number of disinfection	6.9 (±4.7; 5: 4-8.5)	41.9 (±19.5; 41: 29-53)
Volume of ABHR used	26.8 (±17.6; 21.5: 15.6-32.7)	151.8 (±71.1; 164.4: 77.8-214.0)
Volume per action	4.02 (±0.95; 3.73: 3.31-4.52)	**3.63 (±0.87; 3.60: 3.04-4.11)**
P-values comparing after to before		
Time of observation	0.0105	0.196
Number of disinfections	0.399	0.0552
Volume of ABHR used	0.0687	0.0033
Volume per action	0.0117	**0.033**

Note: The average duration of handrubbing monitored by the device was 13.5 sec in 7 HCW who performed a total of 64 actions. The comparison between the time measured by the device and a chronometer evaluated the quality of the time measurement by the device. The chronometer measure had an accuracy of ±1 sec. The mean (±SD, median, maximal negative error, maximal positive error) of the error was -0.13 sec (±1.43, 0.00, -3.0, 3.2

1171
