# Supplementary material for: Effect of Wearing a Novel Electronic Wearable Device on Hand Hygiene Compliance Among Health Care Workers: A Stepped-Wedge Cluster Randomized Clinical Trial
Source: JAMA Netw Open. 2021 Feb 8;4(2):e2035331. doi: 10.1001/jamanetworkopen.2020.35331 (PMC7871189; doi:10.1001/jamanetworkopen.2020.35331)
Supplement: Supplement 2. — eFigure 1. Poster Used to Recruit Participants to the Clinical Trial Aiming to Test a Novel Electronic Wearable Device (SmartRub) eFigure 2. Photograph of SmartRub Device eFigure 3. Starting Dates and Length of Periods of the SmartRub Study (Excluding Wash-out and Follow-up Periods) eTable. HH Practices: Description of Number of Actions, Volume of Applied Alcohol-Based Handrub and Duration of Hand Rubbing, as Well as Overall Correctness of Each Action Per Group and Period as Recorded by SmartRub [file jamanetwopen-e2035331-s002.pdf]

Supplemental Online Content

Pires D, Gayet-Ageron A, Guitart C, et al. Effect of wearing a novel electronic wearable device on hand hygeine compliance among health care workers: a stepped-wedge cluster randomized clinical trial. *JAMA Netw Open*. 2021;4(2):e2035331. doi:10.1001/jamanetworkopen.2020.35331

eFigure 1. Poster Used to Recruit Participants to the Clinical Trial Aiming to Test a Novel Electronic Wearable Device (SmartRub)
eFigure 2. Photograph of SmartRub Device
eFigure 3. Staring Dates and Length of Periods of the SmartRub Study (Excluding Wash-out and Follow-up Periods)
eTable. HH Practices: Description of Number of Actions, Volume of Applied Alcohol-Based Handrub and Duration of Hand Rubbing, as Well as Overall Correctness of Each Action Per Group and Period as Recorded by SmartRub

This supplemental material has been provided by the authors to give readers additional information about their work.

© 2021 Pires D et al. *JAMA Network Open.*

eFigure 1. Poster used to recruit participants to the clinical trial aiming to test a novel electronic wearable device (SmartRub®) at the Geriatric Hospital of the University Hospitals of Geneva.

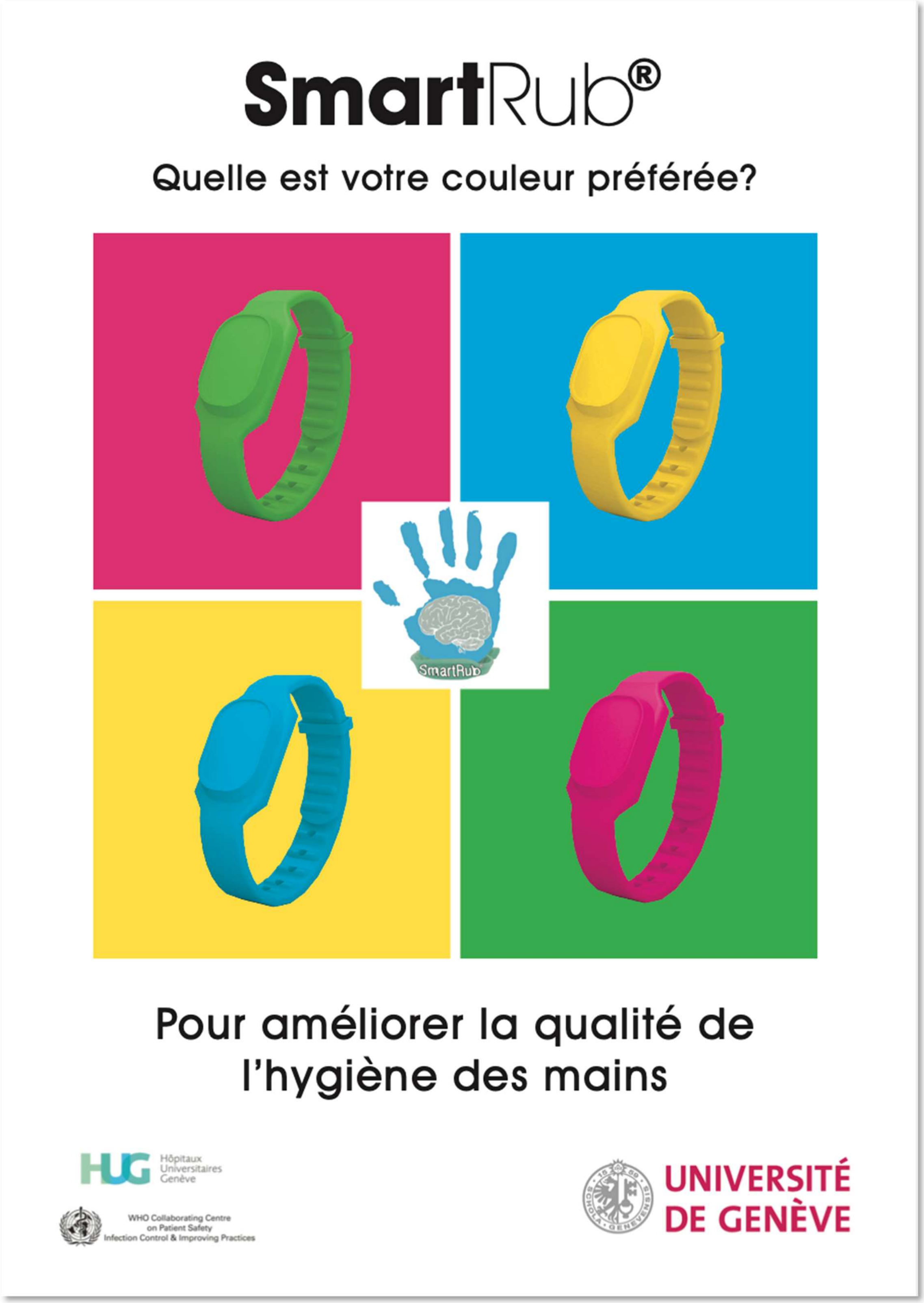

© 2021 Pires D et al. *JAMA Network Open.*

eFigure 2. Photograph of SmartRub Device. The novel electronic wearable device (SmartRub®) consists of two elements, the alcohol-based handrub bottle used at the University Hospitals of Geneva equipped with a volumetric flow meter, and an ergonomic wristband made from medical silicone. The flow meter measured the volume of ABHR poured onto hands and provided feedback by vibrating as soon as the pre-defined hand customized volume had been applied. The wristband measured the duration of each hand hygiene action and vibrated after 15 seconds, independently of the hand rubbing duration performed by the healthcare workers.

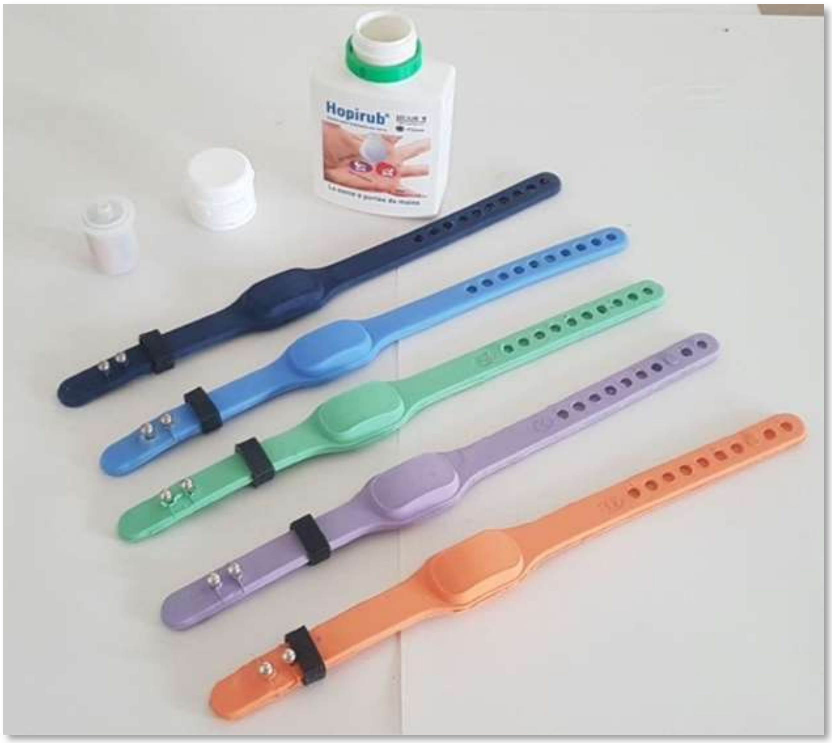

© 2021 Pires D et al. *JAMA Network Open.*

eFigure 3. Starting dates and length of periods of the SmartRub® study (excluding wash-out and follow-up periods). Hand hygiene observations were performed approximately once a month throughout the baseline, transition, intervention and follow-up periods. During the baseline period, healthcare workers did not wear the devices and only hand hygiene observations were performed. During the transition period, the novel electronic wearable device was worn, but the feedback mode (vibration) was not activated, though the device actively monitored the volume of ABHR and duration of hand friction of each HH action. During the intervention period, the feedback (vibration) mode of the device was activated and the monitoring of practices by the device continued. At follow-up, only hand hygiene observations were performed.

After an initial one-month baseline period, wards were randomly assigned to start with the one-month transition period, followed by the intervention period at a rate of three wards per month (from Group1 to Group 4). In total, 12 wards were randomized, following a computer-generated block randomization (1:1:1:1) performed by an independent statistician.

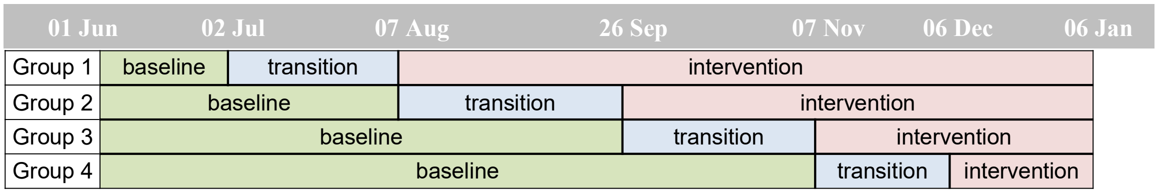

© 2021 Pires D et al. *JAMA Network Open.*

eTable 1. HH practices: description of number of actions, volume of applied alcohol-based handrub and duration of hand rubbing, as well as overall correctness of each action per group and period as recorded by SmartRub®; SmartRub® study, Geriatric Hospital, University of Geneva Hospitals.

	**Group 1 (4 months of intervention)**	**Group 2 (3 months of intervention)**	**Group 3 (2 months of intervention)**	**Group 4 (1 month of intervention)**
Bottle actions (n)				
Transition	10’139	7’319	6’317	2’747
Intervention	22’747	8’573	5’503	2’473
Median volume of applied ABHR per hand rubbing action, mL (IQR)				
Transition	1.02 (0.72-1.49)	1.44 (0.92-2.22)	1.05 (0.78-1.55)	1.03 (0.69-1.56)
Intervention	1.73 (1.03-2.81)	1.86 (1.10-2.93)	1.32 (0.87-2.34)	1.82 (0.87-2.79)
Overall correctness of applied AHBR volume* (95% CI, %)				
Transition	3.7 (3.3-4.1)	18.1 (17.3-19)	11.5 (10.7-12.3)	10.2 (9.1-11.3)
Intervention	30.6 (30-31.2)	32.6 (31.6-33.6)	24.6 (23.4-25.7)	35.3 (33.4-37.2)
Wristband actions (n)				
Transition	8’101	3’160	4’103	1484
Intervention	17’742	5’798	3’560	1865
Median duration of hand rubbing performed per HCWs, seconds (IQR)				
Transition	7 (4.5-11)	6.5 (4.5-11)	5 (4.5-8.5)	7 (4.5-11.5)
Intervention	8 (5-15)	7 (4.5-15)	8 (4.5-15.5)	12 (5.5-17)
Overall correctness of HH duration#, seconds (95% CI, %)				
Transition	12.2 (11.5-13)	15.1 (13.9-16.4)	6 (5.3-6.8)	14.4 (12.6-16.2)
Intervention	25.6 (25-26.3)	25.6 (24.5-26.7)	27.8 (26.4-29.3)	41.1 (38.8-43.3)

*Defined by a volume of ABHR above the estimated hand sized volume.
Defined as at least 15 seconds of hand friction.
ABHR: alcohol-based handrub; CI: confidence interval; IQR: inter-quartile range; HCW: healthcare workers; HH: hand hygiene.

© 2021 Pires D et al. *JAMA Network Open.*
