# Supplementary material for: Effect of Wearing a Novel Electronic Wearable Device on Hand Hygiene Compliance Among Health Care Workers: A Stepped-Wedge Cluster Randomized Clinical Trial
Source: JAMA Netw Open. 2021 Feb 8;4(2):e2035331. doi: 10.1001/jamanetworkopen.2020.35331 (PMC7871189; doi:10.1001/jamanetworkopen.2020.35331)
Supplement: Supplement 3. — Data Sharing Statement [file jamanetwopen-e2035331-s003.pdf]

Data Sharing Statement

Pires. Effect of Wearing a Novel Electronic Wearable Device on Hand Hygiene Compliance Among Health Care Workers. *JAMA Netw Open*. Published February 08, 2021.
doi:10.1001/jamanetworkopen.2020.35331

Data
Data available: No

Additional Information
Explanation for why data not available: We can make the data available upon request.
